# The transcriptional landscape of Shh medulloblastoma

**DOI:** 10.1038/s41467-021-21883-0

**Published:** 2021-03-19

**Authors:** Patryk Skowron, Hamza Farooq, Florence M. G. Cavalli, A. Sorana Morrissy, Michelle Ly, Liam D. Hendrikse, Evan Y. Wang, Haig Djambazian, Helen Zhu, Karen L. Mungall, Quang M. Trinh, Tina Zheng, Shizhong Dai, Ana S. Guerreiro Stucklin, Maria C. Vladoiu, Vernon Fong, Borja L. Holgado, Carolina Nor, Xiaochong Wu, Diala Abd-Rabbo, Pierre Bérubé, Yu Chang Wang, Betty Luu, Raul A. Suarez, Avesta Rastan, Aaron H. Gillmor, John J. Y. Lee, Xiao Yun Zhang, Craig Daniels, Peter Dirks, David Malkin, Eric Bouffet, Uri Tabori, James Loukides, François P. Doz, Franck Bourdeaut, Olivier O. Delattre, Julien Masliah-Planchon, Olivier Ayrault, Seung-Ki Kim, David Meyronet, Wieslawa A. Grajkowska, Carlos G. Carlotti, Carmen de Torres, Jaume Mora, Charles G. Eberhart, Erwin G. Van Meir, Toshihiro Kumabe, Pim J. French, Johan M. Kros, Nada Jabado, Boleslaw Lach, Ian F. Pollack, Ronald L. Hamilton, Amulya A. Nageswara Rao, Caterina Giannini, James M. Olson, László Bognár, Almos Klekner, Karel Zitterbart, Joanna J. Phillips, Reid C. Thompson, Michael K. Cooper, Joshua B. Rubin, Linda M. Liau, Miklós Garami, Peter Hauser, Kay Ka Wai Li, Ho-Keung Ng, Wai Sang Poon, G. Yancey Gillespie, Jennifer A. Chan, Shin Jung, Roger E. McLendon, Eric M. Thompson, David Zagzag, Rajeev Vibhakar, Young Shin Ra, Maria Luisa Garre, Ulrich Schüller, Tomoko Shofuda, Claudia C. Faria, Enrique López-Aguilar, Gelareh Zadeh, Chi-Chung Hui, Vijay Ramaswamy, Swneke D. Bailey, Steven J. Jones, Andrew J. Mungall, Richard A. Moore, John A. Calarco, Lincoln D. Stein, Gary D. Bader, Jüri Reimand, Jiannis Ragoussis, William A. Weiss, Marco A. Marra, Hiromichi Suzuki, Michael D. Taylor

**Affiliations:** 1 Developmental & Stem Cell Biology Program, The Hospital for Sick Children, Toronto, ON Canada; 2 Department of Laboratory Medicine and Pathobiology, University of Toronto, Toronto, ON Canada; 3 The Arthur and Sonia Labatt Brain Tumour Research Centre, The Hospital for Sick Children, Toronto, ON Canada; 4 Department of Biochemistry and Molecular Biology, Cumming School of Medicine, University of Calgary, Calgary, AB Canada; 5 Alberta Children’s Hospital Research Institute, Calgary, AB Canada; 6 Charbonneau Cancer Institute, University of Calgary, Calgary, AB Canada; 7 Department of Medical Biophysics, University of Toronto, Toronto, ON Canada; 8 McGill University Genome Centre, McGill University, Montreal, QC Canada; 9 Department of Human Genetics, McGill University, Montreal, QC Canada; 10 Computational Biology Program, Ontario Institute for Cancer Research, Toronto, ON Canada; 11 Canada’s Michael Smith Genome Sciences Centre, BC Cancer Agency, Vancouver, BC Canada; 12 Department of Neurology, University of California San Francisco, San Francisco, CA United States; 13 Department of Cellular and Molecular Pharmacology, University of California San Francisco, San Francisco, CA United States; 14 Institute of Medical Science, University of Toronto, Toronto, ON Canada; 15 Division of Neurosurgery, The Hospital for Sick Children, Toronto, ON Canada; 16 Department of Molecular Genetics, University of Toronto, Toronto, ON Canada; 17 Division of Haematology/Oncology, Department of Pediatrics, The Hospital for Sick Children, Toronto, ON Canada; 18 SIREDO Center (pediatric, adolescent and young adults oncology), Institut Curie, University of Paris, Paris, France; 19 INSERM U 830, Institut Curie, Paris, France; 20 Unit of Somatic Genetics, Institut Curie, Paris, France; 21 PSL Research University, Université Paris Sud, Université Paris-Saclay, CNRS UMR 3347, INSERM U1021, Institut Curie, Paris, France; 22 Department of Neurosurgery, Division of Pediatric Neurosurgery, Seoul National University Children’s Hospital, Seoul, South Korea; 23 Hospices Civils de Lyon, Institute of Pathology, University Lyon 1, Department of Cancer Cell Plasticity–INSERM U1052 Cancer Research Center of Lyon, Lyon, France; 24 Department of Pathology, The Children’s Memorial Health Institute, Warsaw, Poland; 25 Department of Surgery and Anatomy, Faculty of Medicine of Ribeirão Preto, University of Sao Paulo, São Paulo, Brazil; 26 Developmental Tumor Biology Laboratory, Hospital Sant Joan de Déu, Esplugues de Llobregat, Barcelona, Spain; 27 Departments of Pathology, Ophthalmology and Oncology, John Hopkins University School of Medicine, Baltimore, MD United States; 28 Department of Hematology & Medical Oncology, School of Medicine and Winship Cancer Institute, Emory University, Atlanta, GA United States; 29 Department of Neurosurgery, Kitasato University School of Medicine, Sagamihara, Kanagawa Japan; 30 Department of Neurology, Erasmus University Medical Center, Rotterdam, Netherlands; 31 Department of Pathology, Erasmus University Medical Center, Rotterdam, Netherlands; 32 Division of Experimental Medicine, McGill University, Montreal, QC Canada; 33 Department of Pathology and Molecular Medicine, Division of Anatomical Pathology, McMaster University, Hamilton, ON Canada; 34 Department of Pathology and Laboratory Medicine, Hamilton General Hospital, Hamilton, ON Canada; 35 Department of Neurological Surgery, University of Pittsburgh School of Medicine, Pittsburgh, PA United States; 36 Department of Pathology, University of Pittsburgh School of Medicine, Pittsburgh, PA United States; 37 Division of Pediatric Hematology/Oncology, Mayo Clinic, Rochester, MN United States; 38 Department of Laboratory Medicine and Pathology, Mayo Clinic, Rochester, MN United States; 39 Clinical Research Division, Fred Hutchinson Cancer Research Center, Seattle, WA United States; 40 Department of Neurosurgery, University of Debrecen, Medical and Health Science Centre, Debrecen, Hungary; 41 Department of Pediatric Oncology, Masaryk University School of Medicine, Brno, Czech Republic; 42 Department of Neurological Surgery, University of California San Francisco, San Francisco, CA United States; 43 Department of Pathology, University of California San Francisco, San Francisco, CA United States; 44 Department of Neurological Surgery, Vanderbilt Medical Center, Nashville, TN United States; 45 Department of Neurology, Vanderbilt Medical Center, Nashville, TN United States; 46 Departments of Neuroscience, Washington University School of Medicine in St. Louis, St. Louis, MO United States; 47 Department of Neurosurgery, David Geffen School of Medicine at UCLA, Los Angeles, California, United States; 48 2nd Department of Pediatrics, Semmelweis University, Budapest, Hungary; 49 Department of Anatomical and Cellular Pathology, The Chinese University of Hong Kong, Shatin, New Territories Hong Kong; 50 Department of Surgery, The Chinese University of Hong Kong, Shatin, New Territories Hong Kong; 51 Department of Neurosurgery, University of Alabama at Birmingham, Birmingham, AL United States; 52 Department of Neurosurgery, Chonnam National University Research Institute of Medical Sciences, Chonnam National University Hwasun Hospital and Medical School, Hwasun-gun, Jeollanam-do South Korea; 53 Department of Pathology, Duke University, Durham, NC United States; 54 Department of Neurosurgery, Duke University, Durham, NC United States; 55 Department of Pathology and Neurosurgery, NYU Grossman School of Medicine and NYU Langone Health, New York, NY United States; 56 Department of Pediatrics, University of Colorado Denver, Aurora, CO United States; 57 Department of Neurosurgery, University of Ulsan, Asan Medical Center, Seoul, South Korea; 58 U.O. Neurochirurgia, Istituto Giannina Gaslini, Genova, Italy; 59 Institute of Neuropathology, University Medical Center, Hamburg-Eppendorf, Germany; 60 Research Institute Children’s Cancer Center, Hamburg, Germany; 61 Pediatric Hematology and Oncology, University Medical Center, Hamburg-Eppendorf, Germany; 62 Division of Stem Cell Research, Institute for Clinical Research, Osaka National Hospital, Osaka, Japan; 63 Division of Neurosurgery, Centro Hospitalar Lisboa Norte (CHULN), Hospital de Santa Maria, Lisbon, Portugal; 64 Instituto de Medicina Molecular João Lobo Antunes, Faculdade de Medicina, Universidade de Lisboa, Lisbon, Portugal; 65 Division of Pediatric Hematology/Oncology, Hospital Pediatría Centro Médico Nacional century XXI, Mexico City, Mexico; 66 Division of Neurosurgery, Toronto Western Hospital, University Health Network, Toronto, ON Canada; 67 MacFeeters-Hamilton Center for Neuro-Oncology Research, Princess Margaret Cancer Centre, University Health Network, Toronto, ON Canada; 68 Department of Surgery, Division of Thoracic and Upper Gastrointestinal Surgery, Faculty of Medicine, McGill University, Montreal, QC Canada; 69 Cancer Research Program, Research Institute of the McGill University Health Centre, Montreal, QC Canada; 70 Department of Medical Genetics, University of British Columbia, Vancouver, BC Canada; 71 Department of Molecular Biology & Biochemistry, Simon Fraser University, Burnaby, BC Canada; 72 Department of Cell & Systems Biology, University of Toronto, Toronto, ON Canada; 73 Adaptive Oncology, Ontario Institute for Cancer Research, Toronto, ON Canada; 74 The Donnelly Centre, University of Toronto, Toronto, ON Canada; 75 Department of Pediatrics, University of California San Francisco, San Francisco, CA United States; 76 Department of Surgery, University of Toronto, Toronto, ON Canada

**Keywords:** CNS cancer, Paediatric cancer, Genome informatics, Genetics research

## Abstract

Sonic hedgehog medulloblastoma encompasses a clinically and molecularly diverse group of cancers of the developing central nervous system. Here, we use unbiased sequencing of the transcriptome across a large cohort of 250 tumors to reveal differences among molecular subtypes of the disease, and demonstrate the previously unappreciated importance of non-coding RNA transcripts. We identify alterations within the cAMP dependent pathway (*GNAS*, *PRKAR1A*) which converge on *GLI2* activity and show that 18% of tumors have a genetic event that directly targets the abundance and/or stability of MYCN. Furthermore, we discover an extensive network of fusions in focally amplified regions encompassing *GLI2*, and several loss-of-function fusions in tumor suppressor genes *PTCH1*, *SUFU* and *NCOR1*. Molecular convergence on a subset of genes by nucleotide variants, copy number aberrations, and gene fusions highlight the key roles of specific pathways in the pathogenesis of Sonic hedgehog medulloblastoma and open up opportunities for therapeutic intervention.

## Abstract (web summary)

Sonic Hedgehog medulloblastoma (Shh-MB) comprises four subtypes each with distinct clinical traits. Here the authors characterize the genome, transcriptome, and methylome of Shh-MB subtypes, revealing a complex fusion landscape and the molecular convergence of MYCN and cAMP signaling pathways.

## Introduction

Medulloblastoma (MB) is the most common malignant pediatric brain tumor and a major cause of morbidity and mortality in the pediatric population^1^. Current therapy consists of maximal safe resection, radiotherapy in patients over 36 months, and cytotoxic chemotherapy. MB is thought to comprise a group of four molecularly distinct diseases: Wnt, Sonic Hedgehog (Shh), Group 3, and Group 4^2^. Shh-MB is clinically heterogeneous with infants, teenagers and adults affected. Shh-MB likely comprises four molecular subtypes, Shh-α (adolescents), Shh-β (babies with a poor prognosis), Shh-γ (babies with a good prognosis), and Shh-δ (adults)^3^. The vast difference in the host (babies versus adolescents versus adults) dictates different treatment approaches for different molecular subtypes. Prior delineation of Shh-MB subtypes used expression microarrays^4^, and/or DNA methylation arrays^3^, and the biology underlying the differences among the subtypes is poorly understood.

To further understand the biology of Shh-MB and its molecular subtypes, we studied 250 human Shh-MB using strand-specific RNA sequencing with the incorporation of DNA methylation, whole-genome sequencing, and SNP 6.0 copy number analysis. This non-biased approach to the Shh-MB transcriptome allows us to understand the transcriptional basis and underlying biology of Shh-MB and reveals a previously unsuspected role for many non-coding RNAs. We find disruption in the cAMP pathway converging on Shh signaling and also detect a cluster of mutations in *MYCN* which prevent degradation by FBXW7. Alterations in these genes are mutually exclusive of each other and found in 18% of Shh-MB tumors. We also identify a number of fusion transcripts in Shh-MB, many of which fall within focally amplified regions and known Shh-MB tumor suppressors. This analysis of a large cohort of similar tumors highlights previously unsuspected examples of molecular convergence where the same gene or pathway is activated through diverse molecular mechanisms, emphasizing the importance of those drivers in Shh-MB. Genetic events in Shh-MB do not assort randomly across the cohort, but rather show very restricted patterns of mutual exclusivity, suggesting specific biology, with implications for Shh-MB modeling, and perhaps for the design of synthetic lethal approaches to therapy.

## Results

### Importance of the non-coding transcriptome in Shh-MB

**Fig. 1:**
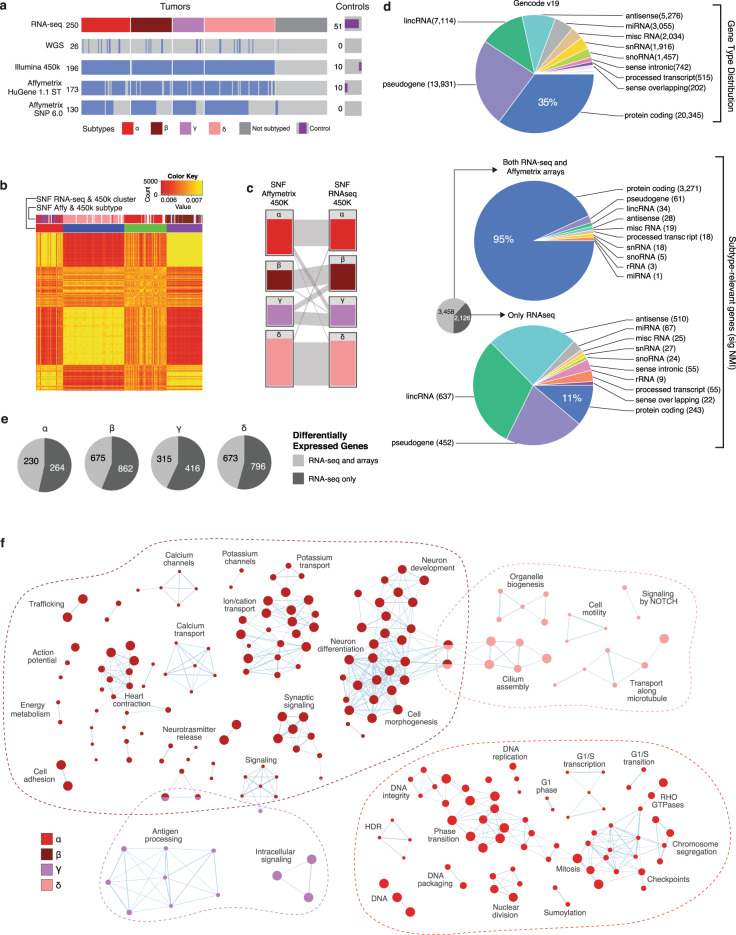
Importance of the non-coding transcriptome in Shh-MB. **a** Overview of Shh-MB RNA-seq samples and overlapping data sources. **b** Heatmap of the sample-to-sample fused network (SNF) by cluster (*k* = 4, *n* = 250). Sample similarity is represented by red (less similar) to yellow (more similar) coloring inside the heatmap. **c** Subtype clusters obtained by Similar Network Fusion (*k* = 4) using Affymetrix expression + 450 K methylation and RNA-seq + 450 K methylation (*n* = 196). Relationships between clustering methods are indicated by gray bars between columns. **d** Biotype distribution amongst all genes (top) as compared to genes that differentiate subtypes (significant normalized mutual information (NMI) from SNF RNA-seq + 450 K methylation), in both RNA-seq and microarray datasets (middle) or restricted to only the RNA-seq dataset (bottom). **e** Differentially expressed genes per subtype (RNA-seq). Genes found only with RNA-seq data are indicated. **f** Enrichment map of biological processes and pathways in Shh-MB subtypes. Each node represents a pathway or process and connecting lines represent common genes between them. Nodes with many shared genes are grouped together and labeled with a biological theme. The color of the nodes refers to the subtype(s) in which the process is enriched. The size of the node is proportional to the number of genes in the process.

Our Shh-MB strand-specific RNA-seq samples (*n* = 250) were additionally characterized with whole-genome sequencing (WGS) (*n* = 26), Infinium Human Methylation 450 K BeadChip (*n* = 196), Affymetrix HuGene 1.1 expression arrays (*n* = 173), and Affymetrix SNP 6.0 arrays (*n* = 130) (Fig. 1a; Supplementary Data 1). Integrative analysis and unsupervised clustering of both RNA-seq and 450 K methylation data allowed us to assign Shh-MB samples to their appropriate molecular subtype^3^. Subtype assignment based on RNA-seq and 450 K methylation data highly overlap with subtyping using Affymetrix expression and 450 K methylation arrays (Fig. 1b, c). While protein-coding genes make up only 35% of the transcriptome in GENCODE (v19), 95% of subtype-specific genes identified using expression arrays are protein-coding (Fig. 1d). However, Shh-MB subtype-specific transcripts identified with RNA-seq encompass many non-coding RNA species, including long non-coding RNAs, expressed pseudogenes, and microRNAs (Fig. 1d; Supplementary Data 2). Indeed, the majority of genes differentially expressed between subtypes using RNA-seq data are non-coding transcripts, which are not evaluated by expression arrays (Fig. 1e). While many of these non-protein-coding genes are poorly annotated, pathway analysis reveals divergent biological mechanisms among Shh-MB subtypes (Fig. 1f). We conclude that each Shh-MB subtype has a unique landscape of non-coding transcripts which may play an important role in the biology of Shh-MB.

### cAMP-dependent pathway alterations converge on GLI2 activity

**Fig. 2:**
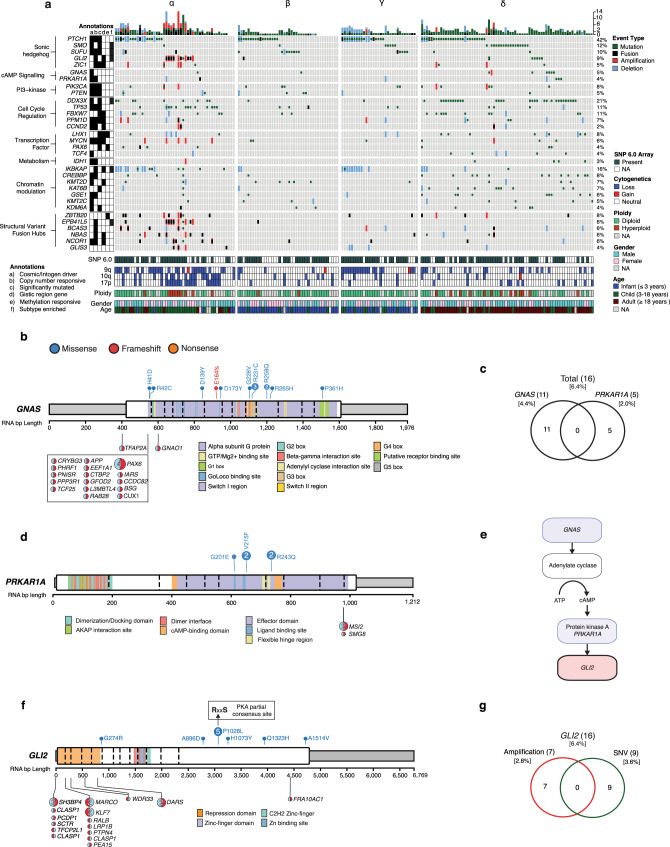
cAMP-dependent pathway alterations converge on GLI2 activity. **a** Oncoprint summaries of all fusion, mutation, and copy number data (*n* = 196). Subtypes are denoted above. NA, not available. **b** Gene-level summary of *GNAS* events. **c** Mutual exclusivity of *GNAS* and *PRKAR1A* LOF events. LOF events include mutations and homozygous deletions. **d** Gene-level summary of *PRKAR1A* events. **e** cAMP dependant signaling pathway schematic. Red indicates activating alterations while blue indicates inactivating alterations. **f**, **g** Gene-level summary of (**f**) *GLI2* events and (**g**) their overlaps. Mutations in **f** are shown as lollipop diagrams above the gene schematic and fusion events are shown below. The 5 prime and 3 prime orientation of the fusion transcript is indicated by the color orientation. In cases where *GLI2* is the 3 prime partners, the fusion lollipop is red on the right.

We investigated the incidence and patterns of mutations in a subtype-specific manner (Fig. 2a; Supplementary Data 3). We detect mutations in *GNAS*, a heterotrimeric Gs protein α subunit (Gαs), in 4.4% of Shh-MB. Most mutations cluster between the GTPase and helical domains which are predicted to reduce GTP binding (Fig. 2b). GNAS activates adenylyl cyclase which increases intracellular cAMP, there-by activating protein kinase A (PKA), a negative regulator of the Shh signaling pathway. This is in line with the phenotype of *Gnas* knockout mice which develop Shh-MBs^5^. Direct phosphorylation of *GLI2* by the PKA complex leads to proteolytic conversion of *GLI2* into its repressor form and abrogation of Shh target gene expression. Correspondingly, we also observe mutations mutually exclusive of *GNAS* in *PRKAR1A*, a critical component of the PKA complex (Fig. 2c, d). All *PRKAR1A* mutations localize to the binding pocket of the cAMP-binding domain impairing the activation of PKA^6^. Nearly all patients with alterations in *GNAS* or *PRKAR1A* do not have any alterations in the Shh signaling pathway (i.e., *PTCH1*, *SMO*, *SUFU*, *GLI2*) (*P* = 3.80 × 10^−5^; two-sided Fisher’s exact test), suggesting that aberration of the cAMP-dependent pathway can lead to Shh pathway activation (Fig. 2e). Single nucleotide variants (SNVs) were also found in *GLI2* within the activation domain^7^ (Fig. 2f) which are largely exclusive of *GLI2* amplification or fusions (Fig. 2g). Most recurrent is the p.P1028L mutation found within a partial PKA consensus sequence^8^, which may interfere with phosphorylation and prevent conversion into its repressor form. Other SNVs can disrupt binding to *SUFU* (p.G274R). Interestingly, nearly all patients with mutations in *GLI2* had no other alterations in Shh pathway constituents (*PTCH1*, *SMO*, *SUFU*) (*P* = 0.015; two-sided Fisher’s exact test) further suggesting an oncogenic role. In conclusion, we describe an alternative axis of control for the Shh-signaling pathway and open up more opportunities for therapy through activation of cAMP signaling.

### Alterations in cell cycle control genes

**Fig. 3:**
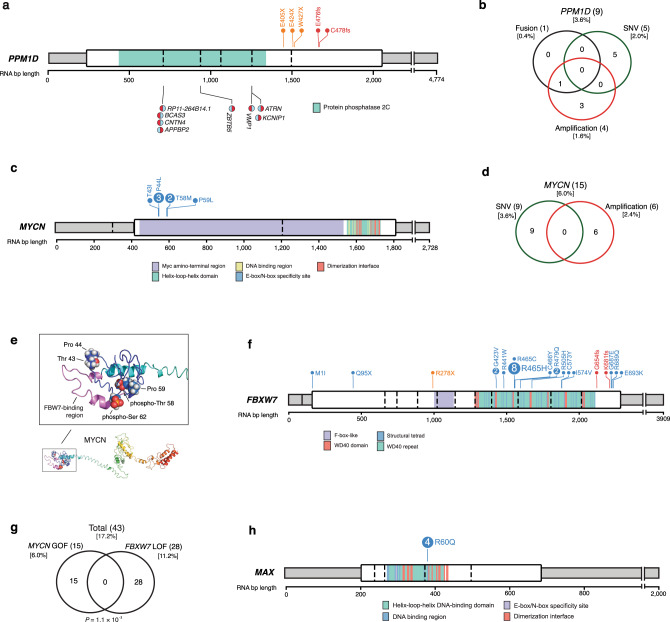
Alterations in cell cycle control genes. **a**, **b** Gene-level summary of (**a**) *PPM1D* events and (**b**) their overlaps. **c** Gene-level summary of *MYCN* events. Only mutations in the canonical isoform NM_005378 are shown. **d** Overlap of *MYCN* fusion, amplification, and SNV events. **e** Structural model of MYCN highlighting positions affected by hotspot mutations (blue) near the FBWX7 protein binding region (purple), and phospho-degron positions (red). **f** Gene-level summary of *FBXW7* events. **g** Mutual exclusivity of *MYCN* gain-of-function (GOF) and *FBXW7* loss-of-function (LOF) events. *P*-value calculated using the DISCOVER package. GOF and LOF events include both high-level CNA and mutation events. **h** Gene-level summary of *MAX* events.

Several Shh-MB drivers important for cell cycle control which were previously identified as amplified, (i.e., *MYCN* and *PPM1D*) also harbor damaging mutations in a subset of patients. *PPM1D*, a negative regulator of the p53 DNA damage response pathway^9^ undergoes nonsense and frameshift mutations at its C-terminus (Fig. 3a, b), all of which are predicted to leave its phosphatase activity intact while significantly increasing protein stability^10–12^. We also detect a cluster of SNVs in *MYCN* within the phospho-degron containing MBI domain (Fig. 3c). *MYCN* amplifications and SNVs are mutually exclusive (Fig. 3d). Phosphorylation of MYCN at S62 primes for second phosphorylation at T58 by glycogen synthase kinase-3 (GSK3). Subsequent dephosphorylation at S62 leads to recruitment of the FBXW7 E3 ubiquitin ligase complex to a phosphodegron motif that includes amino acids both N-terminal and C-terminal to pT58^13^, and the consequent ubiquitination of MYCN^14,15^. Mutations in this region of MYCN disrupt FBXW7 binding and/or ubiquitination, and are predicted to stabilize MYCN^16^ (Fig. 3e). Remarkably, we also identify missense mutations of *FBXW7* within tryptophan-aspartic acid motif (WD40) (Fig. 3f, g)^17–20^ that binds MYCN, in >10% of Shh-MB, which are mutually exclusive of *MYCN* amplification or SNVs. Finally, we found a mutational hotspot (p.R60Q) in the MYC heterodimer partner MAX (1.6% of Shh-MB tumors) (Fig. 3h). These alterations lie within the bHLH-Zip domain involved in protein–protein interactions and DNA binding and may upregulate MYC activity^21^. In conclusion, we find that 18% of Shh-MB patients have a genetic event that directly targets the abundance and/or stability of MYCN.

### Somatic copy number aberrations in Shh-MB

**Fig. 4:**
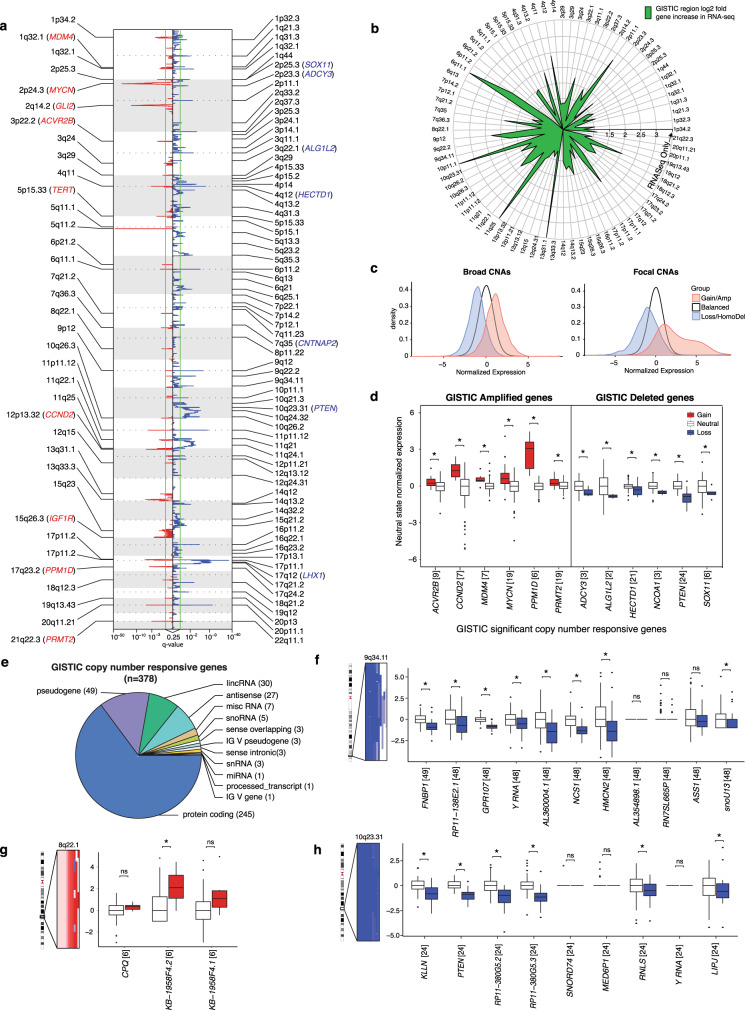
Somatic copy number aberrations in Shh-MB. **a** GISTIC significant amplifications (red) and deletions (blue) observed in Shh-MB (*n* = 126). **b** Log2 fold increase of known annotated gene in GISTIC regions using RNA-seq compared to expression arrays. GISTIC regions with genes only found in the RNA-seq dataset have points on the outermost circle. **c** Normalized expression density across broad and focal CNAs. **d** Expression difference between copy number neutral and aberrant states in GISTIC region copy number responsive genes. Each gene was normalized by its neutral copy number state distribution. Numbers in square brackets denote the number of patients detected with the CNA. The lower and upper hinges in the boxplot correspond to the first and third quartiles while the center line represents the median. The upper and lower whisker extends from the nearest hinge to the smallest/largest value at most 1.5 times the interquartile range. Points outside this range are outliers and are plotted individually. **e** GISTIC copy number responsive gene types. **f**–**h** Expression difference between copy number neutral and aberrant states in (**f**) 9q34.11, (**g**) 8q22.1, and (**h**) 10q23.31. Asterisks annotate significant copy number responsive genes (FDR < 0.05) calculated using a Kruskal-Wallis rank-sum test. Please refer to Supplementary Data 5 for exact *P*-values. The SNP 6.0 copy number segments are shown to the left of each graph. The expression of each gene was normalized by the expression median of the neutral copy number state.

Regions of recurrent genomic gain and loss identify both known Shh-MB driver genes (i.e., *MYCN*, *GLI2*, *PPM1D*, *PTEN*)^22^, as well as the putative drivers (i.e., *PRMT2*, *HECTD1*, *SOX11*, and *LHX1*) (Fig. 4a; Supplementary Data 4). Several recurrent somatic copy number aberrations (CNAs) that do not contain any genes when studied by expression arrays, do contain transcripts when studied by RNA-sequencing (Fig. 4b). Regions of focal amplification are much more likely to show concomitant changes in gene transcription as compared to larger, broad copy number changes (Fig. 4c). A number of putative Shh-MB driver genes encompassed by focal gains or deletions demonstrate copy number-driven expression, further supporting their role as drivers (Fig. 4d; Supplementary Data 5). Notably, only 15% (378/2,536) of genes identified within GISTIC regions show copy number-driven expression (Fig. 4e, Supplementary Fig. 1A–C). In many cases, the copy number responsive genes are poorly annotated non-coding RNAs that might first be overlooked (Fig. 4e−h, Supplementary Fig. 1D−F). We also observe significant deletions in 9q34.11 encompassing the copy number responsive gene *GPR107* (Fig. 4f). This region is usually lost in the context of chromosome 9q loss along with *PTCH1* and *IKBKAP* (Supplementary Fig. 1G, H). A substantial minority (24%) of Shh-MB are aneuploid; their transcriptome differs from diploid tumors by over-expression of genes involved in RNA processing and translation (Supplementary Fig. 2A−D). We conclude that regions of focal CNAs in the Shh-MB genome contain both copy number responsive and non-responsive genes, that many events focus on poorly characterized non-coding transcripts, and that non-copy number responsive genes within CNAs are likely to a poor choice for the development of targeted therapy.

### Identification of Shh-MB fusion genes

We identified fusion transcripts in the Shh-MB transcriptome using three distinct assembly and alignment-based callers (STAR-fusion, InFusion, Trans-Abyss)^23–25^, filtering out any readthrough transcripts or fusion contigs that were also observed in libraries of non-cancerous brain tissue (Supplementary Figs. 3 and 4; Supplementary Data 6). A subset of Shh-MB patients (12/126, 10%) harbor a high number (top 25th percentile) of both fusions and copy number events and are significantly associated with both aneuploidies (10/12; *P* = 7.4 × 10^−7^, two-sided Fisher’s exact test) and with *TP53* mutation (6/12; *P* = 1.2 × 10^−4^, two-sided Fisher’s exact test) (Supplementary Fig. 5A). Only a subset of fusion transcripts demonstrates substantial evidence of an underlying structural variant (SV) in the genome due to the presence of breakpoints in matching WGS or SNP 6.0 data and/or the identification of multiple splice variants of the same fusion transcript. The number of SV-supported fusions per patient was significantly different among subtypes (*P* = 4.7 × 10^−8^; Kruskal-Wallis rank-sum test), with Shh-α showing the highest number of fusions per tumor (Supplementary Data 6).

**Fig. 5:**
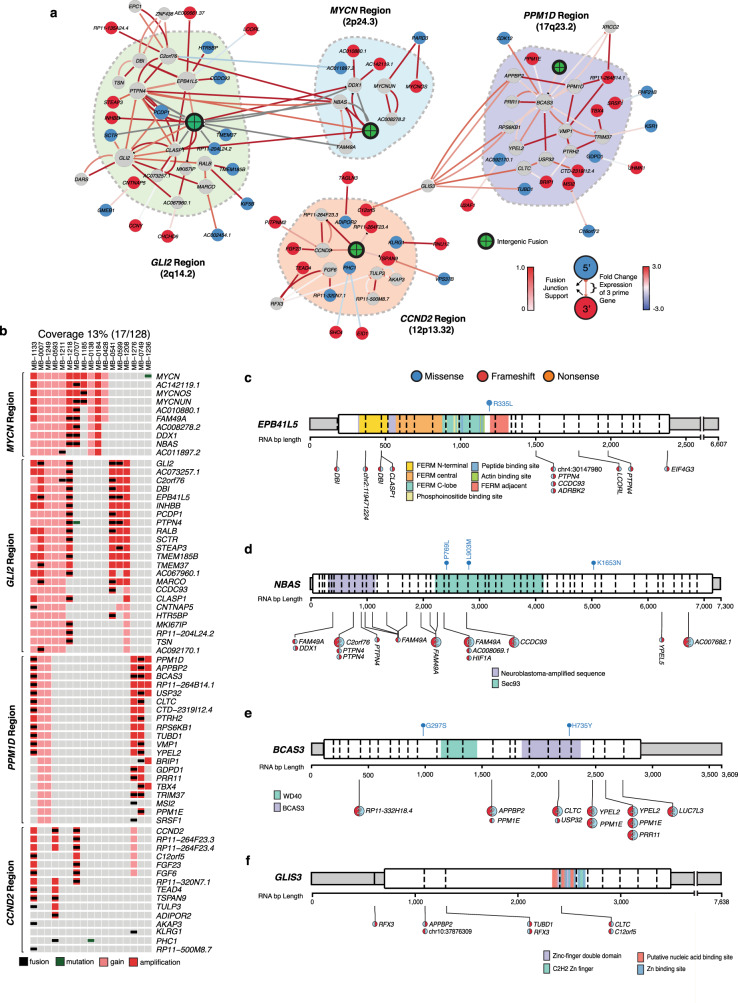
Fusion networks within somatic recurrently amplified regions. **a** The network of gene fusions in focally amplified regions. Node color signifies the most common orientation of the fusion gene, 5 prime (blue), 3 prime (red), or both (gray). The arrow and base color show the proportion of chimeric reads compared to wildtype supporting the fusion. The arrow line color shows the difference in expression of the 3 prime fusion partners compared to patients without the detected fusion. **b** Oncoprint of fusions depicted in focally amplified regions illustrated in **a**. **c**–**f** Gene-level summary of (**c**) *EPB41L5*, (**d**) *NBAS*, (**e**) *BCAS3*, and (**f**) *GLIS3* events. Refer to Fig. 2b for schema description.

**Fig. 6:**
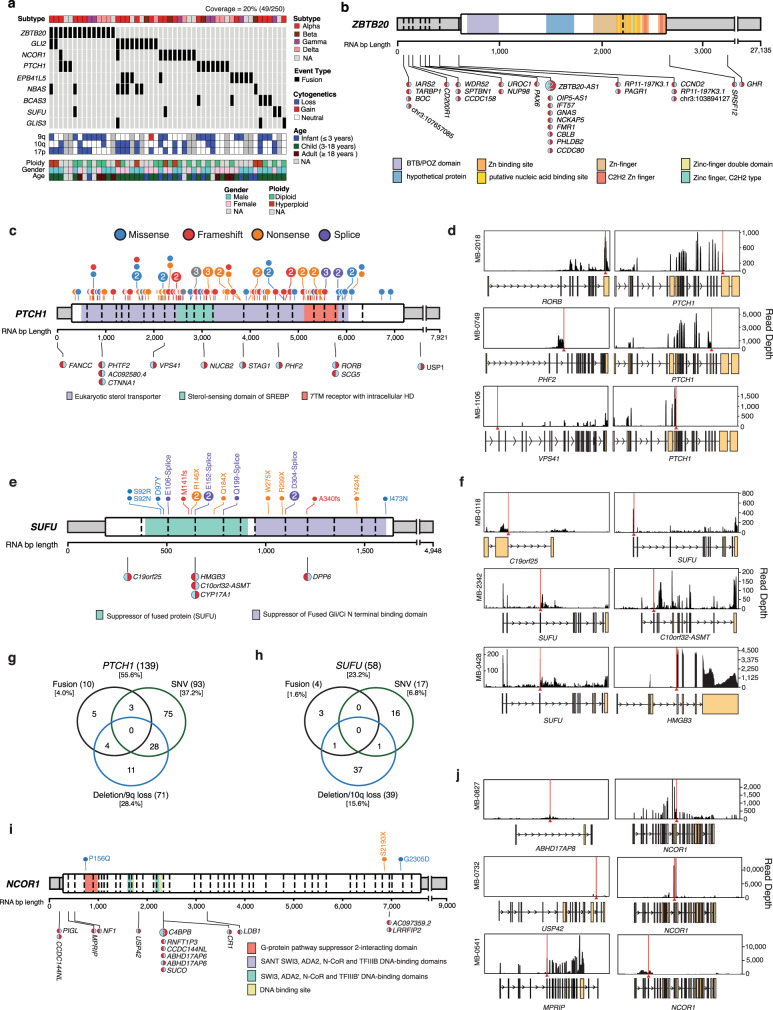
Recurrent fusions in Shh-MB. **a** Oncoprint of fusions detected in focally amplified regions and known Shh-MB tumor suppressors. NA, not available. **b** Gene-level summary of *ZBTB20* events. Mutations are shown as lollipop diagrams above the gene schematic and fusion events are shown below. The 5 prime and 3 prime orientation of the fusion transcript is indicated by the color orientation. In cases where *ZBTB20* is the 3 prime partners, the fusion lollipop is red on the right. **c** Gene-level summary of *PTCH1* events. **d** Read depth diagrams of representative *PTCH1* fusion events. **e** Gene-level summary *SUFU* events. **f** Read depth diagrams of representative *SUFU* fusion events. **g** Overlap of *PTCH1* fusion, amplification, and mutation events. **h** Overlap of *SUFU* fusion, amplification, and mutation events. **I** Gene-level summary of *NCOR1* events. **j** Read depth diagrams of representative *NCOR1* fusion events.

A large number of SV-supported fusions coincide with focal amplification of *GLI2* (2q14.2), *MYCN* (2p24.3), *CCND2* (12p13.32), and *PPM1D* (17q23.2) (Fig. 5a, b; Supplementary Fig. 5B−G). Most recurrently, we observe *GLI2* fusion transcripts (11/250 Shh-MB) fused in the 5 prime ends of the mRNA which houses the repressor domain of the encoded protein, suggesting that the fusion leads to an overactive protein (Fig. 2f). *GLI2* fusions were largely exclusive of detected SNV events and were also found in patients without *GLI2* amplifications (Fig. 2a). We additionally observe recurrent fusion transcripts at nearby genomic loci, such as *EPB41L5*, *NBAS*, *BCAS3*, and *GLIS3* which are likely a result of chromothripsis, and/or the formation of extrachromosomal double minutes (Fig. 5c−f)^26,27^. It is unclear the extent to which amplification versus the formation of a fusion transcript contributes to clonal selection (Supplementary Fig. 5B−G), nor is it obvious whether fusion transcripts involving nearby genes are drivers or passengers. Conversely, we now identify fusions in *ZBTB20* (14/250 patients), which are not usually found in the context of amplification (Fig. 6a, b).

We also identify fusion transcripts involving known Shh-MB tumor suppressor genes such as *PTCH1* and *SUFU*, (Fig. 6c–h), both of which are accompanied by decreased expression of the gene immediately following the breakpoint. These are likely markers of chromosomal events that result in loss of gene function and are largely mutually exclusive of tumors with mutations or large chromosomal deletions, supporting their functional role (Fig. 6g, h). We identify N-terminal missense mutations of *SUFU* which are predicted to be damaging, occur in a highly conserved portion of the gene, and are mutually exclusive with mutations in other Shh signaling genes (Fig. 6e). *NCOR1*, a transcriptional regulator of neural stem cell differentiation^28,29^ harbors similar loss-of-function (LOF) fusion transcripts and damaging mutations (13/250, 5.2% of patients) (Fig. 6i, j). We conclude that >20% of Shh-MB patients exhibit fusion transcripts with structural support for an event in the genome.

### The landscape of oncogenic alterations across Shh-MB

**Fig. 7:**
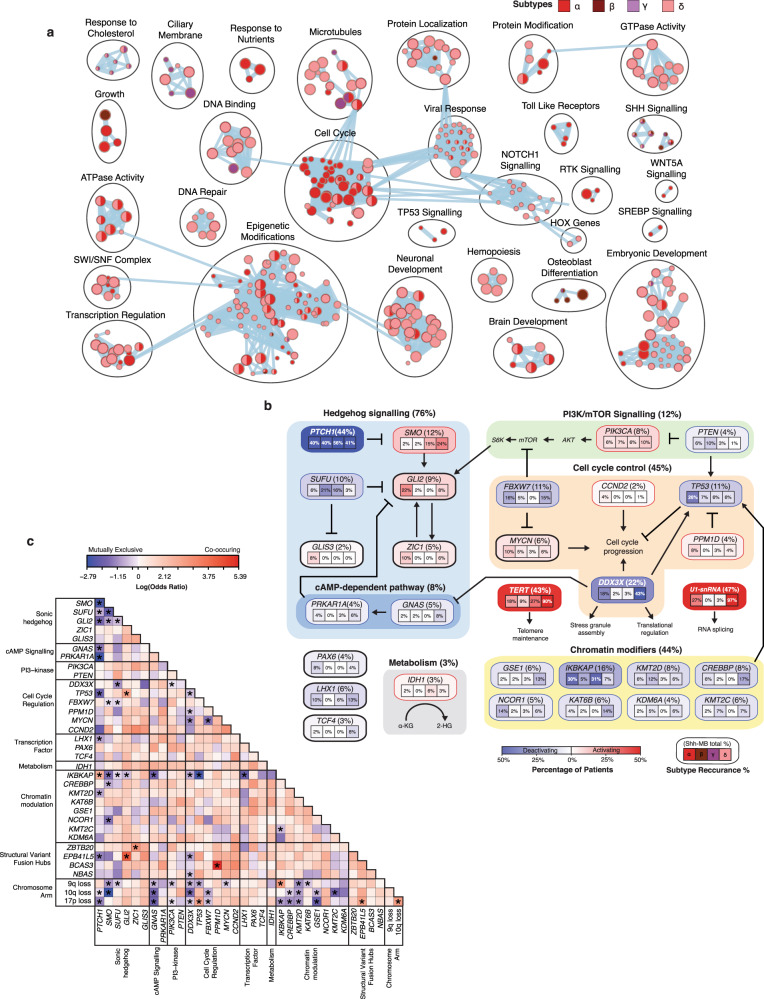
Landscape of oncogenic alterations in Shh-MB. **a** Enrichment map of biological processes and pathways affected by mutation or focal amplifications/deletions in Shh-MB subtypes. Each node represents a pathway or process and connecting lines represent common genes between them. Nodes with many common genes are clustered together and labeled with a biological theme. The node color refers to the subtype(s) in which the process is enriched. The size of the node is proportional to the quantity of genes in the process. Enriched processes were determined using g:Profiler (FDR < 0.05) and visualized in Cytoscape using the Enrichment Map app. **b** Percentage of altered genes and pathways integrating mutation, high-level copy number, and fusion data. Alteration frequencies are expressed as percentages of all cases per subtype (*n* = 196) in the boxes and total percentage across Shh-MB (*n* = 250) in parenthesis beside each gene name. Red indicates activating alterations while blue indicates inactivating alterations. *TERT* and U1-snRNA alternation percentages were obtained from earlier published studies^3,4^. **c** Co-occurrence (red) and mutual exclusivity (blue) among the major Shh-MB driver genes and chromosomal arm events (*n* = 250). Mutually exclusive *P*-values were calculated using the DISCOVER package with FDR < 0.01. Significant Co-occurring genes were found using a two-sided Fisher Exact-Test with FDR < 0.01 and odds ratio >1. Exact *P*-values can be found in Supplementary Data 8.

Transcriptional profiling of this large cohort of a single molecular tumor type permits identification of both recurrent and rare Shh-MB driver genes, and their patterns of mutual exclusivity (Supplementary Data 7 and 8). Most Shh-MBs (86%) have an identifiable event activating the Sonic Hedgehog signaling pathway, including mutations of *PTCH1* (42%), *SMO* (12%), *SUFU* (10%), or *GLI2* (9%) (Fig. 2a). About 11% of patients have previously unappreciated inactivating (i.e., *SUFU* or *PTCH1*), or activating (i.e., *GLI2*) fusion transcripts affecting Shh pathway genes. Pathways discovered using copy number aberrations, mutations, or fusion transcripts were numerous in Shh-α and Shh-δ but limited for Shh-β or Shh-γ due to their low number of mutational events (Fig. 7a). There is strong mutational convergence on genes important for Shh signaling, neuronal development, cell cycle progression, and modification of the epigenome (Fig. 7a, b). Of Shh-MBs without detected events that canonically lead to excess Shh signaling (*PTCH1, SMO, SUFU, TP53, GLI2*, 9q, 10q, and 17p loss) (45/250 patients), the most recurrent mutational events involved *DDX3X* (*n* = 12), *KMT2D* (*n* = 6), *PRKAR1A*, *GNAS*, *GSE1* and *CREBBP* (each *n* = 5) (Fig. 2a); all of which have been previously shown to interact with or potentiate Shh signaling^5,30,31^. *DDX3X* and *GSE1* are potent medulloblastoma tumor suppressors in Gorlin 1 NES cells, *PRKAR1A* with its upstream g-protein *GNAS* are both regulators of Shh activity through cAMP, and CREBBP has been shown to promote cell-cycle exit during postnatal development in coordination with Shh pathway upregulation.

We used MethylMix^32^ to identify potential Shh-MB driver genes affected by promoter CpG hypomethylation or hypermethylation, for which there is a correlative change in gene expression (Supplementary Fig. 6). We obtained a curated list of 735 promoter probe-gene pairs (540 and 195 for two and three methylation clusters, respectively), involving 727 genes in total (Supplementary Fig. 6A, B; Supplementary Data 9). Among these, we identify a number of known cancer genes (i.e., *FOXL2*, *RUNX1T1*), transcription factors (i.e., *MEIS2*), as well as *LHX1* and *PAX6 (*which are also recurrently affected by mutations) (Supplementary Data 9). Transcriptional silencing of *PAX6* through promoter CpG methylation, versus somatic mutations of *PAX6,* appear to be largely mutually exclusive (*P* = 7.3 × 10^−4^, multinomial exact test), suggesting convergence on PAX6 loss of function (Supplementary Fig. 6C−H).

Lastly, DISCOVER^33^ was used to identify networks of significantly mutually exclusive genes and chromosome arms across the subgroup and in a subtype-aware manner. We observe extensive significant mutual exclusivity between driver gene pairs in Shh-MB (Fig. 7c; Supplementary Data 8). As expected, the most pronounced negative gene correlations are between members of the Shh signaling pathway (i.e., *PTCH1, SMO, SUFU, GLI2*) (Fig. 7b, c). Chromosomal deletions of 9q, 10q, and 17p seem to be potent drivers, mutually exclusive of genes in the cAMP, Phosphoinositide 3-kinase signaling, cell cycle regulation, and chromatin modulation pathways. All chromosomal losses are significantly mutually exclusive of *GNAS*, *DDX3X*, and *KMT2D*. Furthermore, alterations in *GNAS* and *PRKAR1A* are mutually exclusive of *PTCH1* further supporting its role in upregulating *GLI2* through cAMP dependant signaling. Mutual exclusivity is also observed between *MYCN* and *FBXW7*. We conclude that Shh-MB mutational events exhibit marked patterns of mutual exclusivity which offer insights for modeling of Shh-MB and suggest avenues for synthetic lethal approaches to therapy.

## Discussion

Initial efforts to subdivide cancers through unsupervised clustering primarily used expression microarrays that focused on the protein-coding elements of the genome. Through an unbiased approach using whole transcriptome sequencing, we now identify a large number of non-coding genes as differentially expressed between the molecular subtypes of Shh-MB. This is complementary to our prior discoveries of the most common mutations in Shh-MB, mutations of the *TERT* promoter^34^, and mutations of the U1-snRNA^4^, both of which are non-coding. Assigning biological functions to either individuals or groups of non-coding RNA transcripts is obviously more difficult than it is for protein-coding genes, and thus the importance and specific biological role of most of these differentially expressed non-coding transcripts will need to be addressed in the future through additional functional experiments.

Shh-MBs harbor few mutations, but frequently have more structural and copy number aberrations in their genomes^22^. For many of these CNAs, the specific resident genes driving clonal selection were not previously apparent. Indeed, many of the minimally amplified/deleted intervals appeared to be devoid of transcripts when studied by microarray. Our unbiased transcriptional approach identifies transcripts within almost all intervals and further demonstrates that only a subset of genes within a given region of recurrent CNAs have copy number-driven expression, and thus are possible drivers. Discerning the driver genes within regions of recurrent CNAs might allow for the design of rationally targeted therapies.

Transcriptional profiling of such a large cohort of a single molecular type of cancer allows for a thorough understanding of the tumor’s genomic landscape, including the identification of genes affected by mutations (*GNAS*, *MYCN*, *PPM1D*, and *PRKAR1A*), and fusion transcripts (*ZBTB20* and *NCOR1*). We also report fusion transcripts in known Shh-MB driver genes, that are likely actually tombstones of large genomic events leading to gene inactivation (i.e., *PTCH1*, and *SUFU*). Other drivers previously known to be amplified in Shh-MB are now identified in additional patients as activated through the creation of fusion transcripts (i.e., *GLI2*), and/or point mutations (i.e., *MYCN* and *GLI2*). These latter events in *GLI2* and *MYCN* further support a driver role for these genes in Shh-MB, and are clinically important as their presence in a tumor will likely render them unresponsive to Sonic Hedgehog pathway inhibition using small molecules. Diverse molecular events do appear to converge on a limited set of pathways in Shh-MB, with the different genes showing clear patterns of mutual exclusivity, perhaps telling us about the molecular events that initiate and sustain Shh-MB growth.

## Methods

### Acquisition of patient samples

Samples were obtained from the Medulloblastoma Advanced Genomics International Consortium (MAGIC), and from the International Cancer Genome Consortium (ICGC). All patient material was collected after receiving written informed consent, which includes consent to publish the data, under the ethical regulations of the following institutions: Hospital for Sick Children, Institut Curie Research Center, Université de Lyon, Seoul National University Children’s Hospital, German Cancer Research Center, John Hopkins University School of Medicine, University of São Paulo School of Medicine, Istituto Neurologico Besta, University of Pittsburgh, Emory University, Vanderbilt Medical Center, University of Debrecen Medical and Health Science Centre, Tohoku University, McMaster University, Mayo Clinic, Washington University School of Medicine, St. Louis Children’s Hospital, Seattle Chidren’s Hospital, Fred Hutchinson Cancer Research Centre, Erasmus University Medical Center, University of Warsaw, Children’s Memorial Health Institute, The University of California-San Francisco, The Chinese University of Hong Kong, McGill University Faculty of Medicine, Masaryk University Faculty of Medicine, Hospital Sant Joan de Déu, David Geffen School of Medicine at University of California-Los Angeles, University of Colorado Denver, University of Calgary, University of Ulsan, Asan Medical Center, University of Cincinnati, Cincinnati Children’s Hospital Medical Center, University of Alabama at Birmingham, Universidade de São Paulo-Brazil, UMAE Pediatria-Portugal, Osaka National Hospital, New York University Medical Center, Ludwig Maximilians University, Kolling Institute of Medical Research, Istituto Giannina Gaslini, Duke University, Virginia Commonwealthy University, School of Medicine, University of Nottingham, University of Arkansas, Universitäts Kinderspital, Universitäts Kinderklinik, University Health Network, Semmelweis University, Kumamoto University, Hospital Infantil de Mexico Federico Gomez, and Chonnam National University. Control brain RNA was acquired from commercial suppliers (Brainchain, USA), and control RNA-seq libraries were obtained from the Genotype-Tissue Expression (GTEx) project (phs000424.v7.p2)^35^. Statistical methods were not used to predetermine the study sample size. Only primary Shh-MB samples were selected for this study. The age, gender, subtypes, and available data of the 250 patients used in this study are presented in Supplementary Data 1.

### Sample processing

Samples were obtained fresh from patients at the time of diagnosis and stored at −80 °C. Tissues were either manually homogenized using a mortar and pestle after freezing in liquid nitrogen or processed in an automated manner using a Precellys 24 tissue homogenizer (Bertin Technologies, France), following the manufacturer’s instructions. DNA was extracted by SDS/Proteinase K digestion followed by 2–3 phenol extractions and ethanol precipitation. Total RNA was isolated using the Trizol method (Invitrogen, USA) using standard protocols. DNA and RNA were quantified using a NanoDrop 1000 instrument (Thermo Scientific, USA), and integrity assessed either by agarose gel electrophoresis (DNA) or Agilent 2100 Bioanalyzer (RNA; Agilent, USA) at The Centre for Applied Genomics (TCAG, Toronto, Canada).

### Messenger RNA library construction and sequencing

Total RNA samples (2 µg) were arrayed into 96-well plates, and polyadenylated mRNA was purified with a MultiMACS mRNA isolation kit as per the manufacturer’s instructions. First-strand cDNA was synthesized using a SuperScript cDNA Synthesis kit with random hexamer primers. The SuperScript cDNA Synthesis protocol was used for second-strand cDNA synthesis. dTTP was replaced with dUTP in the dNTP mix which allowed the second strand to be digested with UNG (Uracil-N-Glycosylase, Life Technologies, USA) in the post-adapter ligation reaction. The cDNA was quantified and checked for quality before fragmentation. Plate-based libraries were created following the BC Cancer Agency’s Michael Smith Genome Sciences Centre (BCGSC) paired-end (PE) protocol^36^. The libraries were sequenced using Illumina HiSeq 2000 or 2500, 2 × 100 PE lanes, with v3 chemistry and HiSeq Control Software version 2.0.10.

### Whole-genome library construction

Samples were sequenced on the Illumina HiSeq 2000 or 2500 platform at Canada’s Michael Smith Genome Science Centre in the BC Cancer Agency.

### RNA-seq alignment

The hs37d5 reference genome FASTA (1000 Genomes Project Phase II) was appended to the C1_2 ERCC spike-in sequences used for C1 Fluidigm, as well as Caltech profile 3 spike-ins sequences by ENCODE. A STAR assembly was then built with this reference and GENCODE (v19) gene annotations using parameter ‘*-sjdbOverhang 124*‘. RNA-seq library reads were then mapped with the built assembly using STAR (v2.5.1b) and parameters ‘*-outFilterMultimapNmax 20 -alignSJoverhangMin 8 -alignMatesGapMax 200000 -alignIntronMax 200000 -alignSJDBoverhangMin 10 -alignSJstitchMismatchNmax 5 −1 5 5 -outSAMmultNmax 20 -twopassMode Basic*’.

### Shh-MB subtype identification

The Similarity Network Fusion (SNF) method^37^ was run on 196 primary tumor samples using both RNA-seq gene expression and DNA methylation data to determine Shh-MB subtypes^3^. The full gene expression and methylation matrix were used since the SNF method does not require any prior feature selection. The SNFtool R package (v2.2.0) was used with parameters ‘*K* = *40, alpha* = *0.6, T* = *50’* and then spectral clustering, implemented in the SNFtool package, was run on the SNF fused similarity matrix to obtain the groups corresponding to *k* = 2−12. The four clusters obtained at *k* = 4 corresponded to the four Shh-MB medulloblastoma subtypes, α (*n* = 50), β (*n* = 42), γ (*n* = 32) and δ (*n* = 72).

### Shh-MB subtype relevant genes (NMI)

The Normalized Mutual Information (NMI) score (as part of the SNFtool package) was identified for each feature (i.e., each gene and methylation probe). For each feature, a patient network based on the feature alone was constructed and subsequently used in spectral clustering. This was then compared to the whole fused similarity matrix through the computation of NMI scores^37^. All features were then ranked according to their NMI scores, representing their importance for the fused network (a score of 1 indicates that the network of patients based on the given feature leads to the same groups as the fused network, whereas 0 means no agreement). The top 10% of features (called subtype-relevant genes) were considered for subsequent analysis.

### Shh-MB subtype differentially expressed genes

Differential expression analysis was performed using DESeq2 (v1.24.0) R Bioconductor package^38^ comparing samples from one Shh-MB subtype to the samples from the remaining 3 Shh-MB subtypes, considering significant genes with an FDR < 0.05.

### RNA-seq mutation analysis

RNA-seq mutation calls were performed using GATK (v3.8.0)^39^ using GATK’s best practices and workflows^4^. Detected variants were filtered using a panel of normal controls (9 Brainchain and 42 GTEx RNA-seq libraries), multiallelic mutations, and if candidates had <5 variant reads. Annotation was performed using ANNOVAR software^40^.

Mutations with a frequency greater than 0.01 in 1000 Genomes, dbSNP138, Exome Aggregation Consortium database, NHLBI-ESP project, Kaviar Genomic Variant Database, Haplotype Reference Consortium database, Greater Middle East Variome, Brazilian Genomic Variants database, and from an inhouse SNP database (356 sequenced whole genomes) were discarded. Suspected RNA editing events registered in the RADAR database^41^ were also discarded. Any deletions which were completely matched with an intron registered in the GENCODE (v19) database were also removed since splice junctions caused by canonical splicing were often miscalled as deletions.

Reads were split into intron-exon segments. However, since there remained unsplit-reads overlapping splice junctions, the splice site variant read numbers were re-calculated using a modified ‘realignment’ function of the GenomonMutationFilter package. The default algorithm remapped reads around detected mutations into reference genomic sequences with and without detected variants. Isoform sequences constructed from the GENCODE (v19) database were added, as well as non-annotated isoforms detected using LeafCutter^42^ since Shh-MB often contain U1-snRNA mutations which cause cryptic splicing. Variants on splice sites were calculated using a modified GenomonMutationFilter and any splice sites with < 5 variants were removed.

Candidates on homopolymer sites were filtered out using the following criteria. (1) homopolymer sequence is ≥5 bps, (2) Insertions or deletions, (3) deleted or inserted bases were the same or consecutive base(s) with the homopolymer base. Any mutations only supported by soft-clipped reads were discarded. In addition, SNPs were filtered if: (1) they were present in germline SNP clusters which were defined as any regions ≥10 bps where SNPs were registered on all the positions in dbSNP150. (2) Any missense or synonymous mutations and non-frameshift indels registered in any of the SNP databases listed above and registered with less than 10 samples or, (3) not registered in COSMIC v87. Mutations were also classified as non-pathogenic and removed if: (1) they registered with less than 10 samples in COSMIC v87, (2) the SIFT score ≥0.05, PolyPhen-2 HDIV ≤ 0.908, PolyPhen-2 HVAR ≤ 0.956, “polymorphism” or, (3) “polymorphism_automatic” by MutationTaster^43^, and “predicted non-functional” by MutationAssessor^44^.

Lastly, EBCall^45^ was run using the same normal panel. Candidates with <10^−3^ *P*-value calculated by EBCall were discarded. EBCall uses the samtools mpileup function, so a subset of mutations detected by local-realignment can not be evaluated correctly. Therefore, any mutations which samtools mpileup could call with <5 variant reads, or less than a half of variants reads detected by GATK are not filtered out. Significantly mutated genes (*q* < 0.05) were identified using MutSigCV^46^ with its default setting.

### SNP 6.0 Processing

Affymetrix Power Tools (v1.18.2) was used to process and normalize the probe intensities. The PennCNV-Affy pipeline^47^ was then used to generate the log R ratio (LRR) and B allele frequency (BAF). The probes were mapped onto hg19 using the ‘affygw6.hg19.pfb’ file. All other parameters were left on default.

### Copy number determination and ploidy estimation

The resultant probe level LRR and BAF data were input into ASCAT (v2.4.3)^48^. GC wave correction was performed, followed by germline genotype prediction. Lastly, the ASCAT algorithm was used to find copy number values for each genomic region and the overall ploidy and purity of the sample. Samples, where the model fit was less than 80%, failed the ASCAT processing stage.

### Copy number post processing

The copy number of each segment, as well as the average ploidy of the sample, was used to calculate the log-ratios using the equation: log2((Copy Number)/Ploidy). Adjacent segments whose log-ratios differed by less than 0.25 were merged using the size weighted mean.

### Filtering common variants

To derive filtered lists, the gold standard variants listed in DGV release 2016-05-15 for GRCh37 found in at least 1% of samples were used to remove any segments with a 50% reciprocal overlap with segments produced by ASCAT. Once removed, the remaining segments were merged using their size-weighted means as before. Further filtering was also done using the list variants in the supporting variants list in the DGV release 2016-05-15 for GRCh37. Studies that had at least 50 subjects, as well as variants found in at least 1% of the study, were used, and ASCAT segments that had a reciprocal overlap of 80% with these variants were removed. This was performed after removing variants from the Gold Standard list. The resulting segments were then merged using their size-weighted means. Copy number states were assigned to each segment based on their log ratio and their ploidy values. Segments were then grouped into either broad or focal depending on whether the segment spanned a length greater than 12 Mb, or equal to and less than 12 Mb. These broad and focal segments were then used to determine gene-level states.

### GISTIC analysis and increased genes in RNA-seq

The filtered and size-weighted merged segments were then input into the GISTIC 2.0 module on GenePattern^49^ and run with slight changes to the default parameters: ‘*focal length cutoff* = *0.5, confidence level* = *0.9, q-value* = *0.25, remove X* = *false, run broad analysis* = *yes*‘. The amplified and deleted segments were then extracted from the filtered file and used to determine which genes fell within the region using bedtools (v2.27.1)^50^. Microarray annotations and RNA-seq annotations were used to determine the number of detectable genes captured by each method.

### Gene level determination of copy number state

The copy number segments for each patient were then intersected with the list of GENCODE (v19) genes. The segment that overlapped the greatest amount of the gene was the copy number ratio/state assigned to that gene (e.g., if segment A overlapped with 25% of the gene, while segment B overlapped with 45% of the gene, the gene would be given the ratio/state of segment B. A majority of the gene does not have to be overlapped by a segment to assign it to that ratio/state – similar to “first past the post”). Further to this, for a gene to be gained or amplified, it must overlap at least 50% of the gene, whereas any loss or deletion that overlaps a gene would give that gene this status.

### Copy number responsive gene

Gene expression was categorized based on either having an amplification, neutral or with a loss. The Kruskal-Wallis test was performed on each gene to determine if the gene copy number state corresponded with a significant difference in expression. The significance values were adjusted for multiple testing using the Benjamini-Hochberg method, and genes whose adjusted *P*-values <0.05 were flagged as being copy number responsive.

### Fusion calling

Multiple fusion callers were used to maximizing sensitivity. *Star-Fusion*: STAR RNA-seq read alignment outputs, bam and the ‘Chimeric.out.junction’ file were input into STAR-Fusion^23^ (v0.8.0) using default parameters. STAR fusion results were then further filtered with FusionInspector (v0.8.0) using default settings. *InFusion*: Bowtie2 (v2.2.1)^51^ genome assembly was created using hs37d5 (appended to the C1_2 ERCC spike-in, as well as Caltech profile 3 spike-ins sequences) and GENCODE (v19). Infusion^25^ (v0.7.3) was run twice for each sample, firstly with parameters *‘-allow-intronic -allow-intergenic -allow-non-coding -allow-all-biotypes‘* from which only gene-gene fusions were kept for further filtering. The infusion was run a second time with the addition of more stringent parameters ‘*-min-split-reads 3 -min-span-pairs 2 -min-fragments 4*′, from which only gene-intergenic or intergenic-intergenic fusions were kept. Afterward, both Infusion lists were concatenated. Trans-Abyss: De-novo assembly was conducted using ABySS24 for each RNAseq library^22,24^. Reads were assembled into contigs using different starting k-mer values (substrings of k bp). These contigs were then merged into a smaller non-redundant set. Inter-contig distances were calculated using paired-end information and were used to unambiguously merge contigs. These contigs were then aligned to the reference human genome and known transcripts (UCSC, RefSeq, Ensemble, Aceview). Candidate fusion genes were shortlisted from contigs alignments that matched multiple known annotations and then further analyzed to determine fusion orientation. Predicted fusion contigs were split into two sequences by gene and aligned to the reference (hg19) using BLAT (v35). The predicted orientation was determined to be that which allowed fusion partner genes to be in a sense-sense orientation, similar to what is done in STAR-Fusion. Predicted orientations which were not compatible with both fusion partner genes being in a sense-sense orientation were flagged as low confidence orientations.

### Fusion filtering

A list of blacklisted fusion pairs and breakpoints were created from control GTEx and Brainchain RNA-seq libraries using a (1) fusion contig alignment, and (2) control sample fusion calling strategy: (1) From each detected event, fusion contigs were extracted (110 bp from both the 5 prime and 3 prime partner side where possible) using scripts supplied by the respective fusion caller. These contigs were then used as a reference for alignment of the normal brain RNA-seq libraries using bbmap (v37.33) with parameters ‘*mappedonly semiperfectmode qin* = *33 boundstag* = *t saa* = *f g maxsites* = *1000000 minaveragequality* = *30 ambiguous* = *all*‘. A fusion was blacklisted if a high-quality control sample read (bp quality average >30) aligned perfectly with the fusion contig with at least a 20 bp overhang past the fusion junction. If the same fusion gene-pair was found in ≥2 control samples, it was also subsequently blacklisted. (2) STAR, InFusion, and Trans-Abyss fusion callers were used on all fetal and adult control brain samples using the same parameters as the tumor libraries. Any fusion pairs detected in the fetal MAGIC control and at least 2 adult samples were blacklisted. Furthermore, all fusion breakpoints detected in any control samples callers were blacklisted.

Any fusions in the control sample breakpoint and gene pair blacklists were filtered out, as well as fusions where both fusion breakpoints were called within the same gene (circular RNA artifacts). In an effort to minimize the number of readthrough fusions, fusion pairs within 50 kb and fusions with highly recurrent breakpoints (>15 samples with the same event) were filtered out unless there were other fusion breakpoints detected in the same genes. Highly expressed genes often contained readthrough fusions so the ratio of ((fusion reads)/200 bp)/(gene RPKM) was calculated and any fusions where either partner had a ratio of <0.01 were removed. Fusions, where the read proportion supporting the fusion junction was less than 0.05 for both partners, were also removed. From this filtered list, an event was further characterized as a structural variant (SV) based fusion if it was validated by WGS or SNP 6.0 (see Fusion validation method), or if there were multiple fusion isoforms detected with both spanning reads and bridging reads >0 and spanning + bridging sum >20 in at least one partner. For highly recurrent fusion genes, the unfiltered events were manually inspected and salvaged if there was a change in reading depth at the fusion junction or WGS/SNP 6.0 support. Gviz (v1.18.2)^52^ was used to visualize the change in reading depth associated with each fusion event.

### Fusion validation

#### WGS

There were different assigned validation states based on the location of the two partner genes relative to the location of WGS detected breakpoints: (1) fused exon is first or last exon and the breakpoint falls into the intergenic region between the gene and adjacent gene, (2) fused exon is the middle exon and the WGS breakpoint falls within an adjacent intron (3) breakpoint falls within a 100 kbp window from the edge of the fused exon. Confidence levels were assigned as follows: High-Both partner genes meet conditions (1) or (2), Intermediate-One partner meets condition (1) or (2) and the other partner fulfilled (3), Low-Both partners meet condition (3).

#### SNP 6.0

The position of RNA fusion breakpoints was compared to SNP 6.0 predicted breakpoints corresponding to a change in copy number. The SNP 6.0 breakpoints were padded with a 250 kbp window upstream and downstream, and then each RNA fusion breakpoint in a pair was checked for support (i.e., support for each breakpoint of a fusion was done, respectively) using bedtools (v2.27.1). The support of each fusion was reported as left-sided (only the first breakpoint of the fusion was detected), right-sided (only the second breakpoint of the fusion was detected), both (both breakpoints of the fusion were detected), or none.

### WGS alignment

Whole-genome sequencing reads were aligned to the human reference genome “hs37d5” by 1000 Genomes Project Phase II using Burrows-Wheeler Aligner (BWA)-MEM, (v0.7.8) with ‘-T 0’ parameter. Duplicates were marked using biobambam (v0.0.148)^53^.

### WGS structural variant calling

Somatic structural variant calling was performed using two softwares: Genomon-SV (v0.4.1)^54^ and DELLY2 (v0.7.5)^55^. Genomon-SV was run using its default settings. Detected candidates were filtered with *‘-min_tumor_allele_freq 0.02 -max_control_variant_read_pair 1 -control_depth_thres 10 -inversion_size_thres 1000 -min_overhang_size 50 -remove_simple_repeat’*. DELLY2 was also run using its default settings. The following filter was used for somatic structural calls: *‘-m 15 -a 0.1’* for deletion, *‘-m 400 -a 0.1’* for tandem duplication and inversion, *‘-m 0 -a 0.1’* for translocation. DELLY2 results were filtered using 341 control whole-genome sequence data using ‘filter’ function of DELLY2 with its default setting. Both results were merged and detected candidate mutations were reanalyzed using velvet de novo assembler^56^. Soft-clipped and one-anchor reads were extracted within 1000 bp of detected breakpoints from the tumor and matched control whole genome sequence. Then, contigs were generated using velvet with *‘-short’* option and hash length *‘11, 72, 10’* (from 11 to 72 with a step of 10). Reference sequences were prepared for remapping which contained reference sequences ±1200 bp around both paired breakpoints and expected variant sequences with the somatic structural variant. Contigs were mapped to the references using blat version 35 with *‘-fine’* function. Only the candidates where contigs from tumor were mapped on the variant sequences and not found mapped in the control were used.

### MYCN protein structural model

To predict protein structure, the weighted existing structural information of some MYCN and MYC regions from the RSCB PDB (5G1X, 6G6J, 1NKP, 2A93) were used in i-TASSER^57,58^. These models were subsequently visualized and modified in PyMOL (v2.3) and UCSF Chimera (v1.13.1)^59^. The prediction is imprecise, as the structure of the N-terminus of MYCN shows intrinsic disorder.

### Mutual and co-occurrence analysis

Both the DISCOVER^33^ R package (v1.1.0) and a Fisher exact test were used to calculate mutual exclusivity and co-occurrence on high-level copy number, mutation, SV fusion events, and arm level gains/losses using default parameters on all patients and on a per-subtype basis. Only known drivers, significantly mutated, GISTIC copy number responsive genes, and arm level events (*n* = 384) were included and a corrected *P*-value < 0.01 was used for downstream analysis. Both the Fisher and DISCOVER *P*-values were corrected using the false discovery rate.

### Pathway analysis

#### Subtype driving genes

Enriched pathways were identified using the gProfileR R package^60^. Four gene lists corresponding to the four Shh-MB subgroups were generated by selecting the top 10% genes having the highest NMI scores and a positive *Z*-score. Each gene list was ranked by *Z*-scores in decreasing order and analyzed by the gProfileR function with the ordered query setting. Pathways from the Reactome pathway database and biological processes (BP) from Gene Ontology that have between 5 and 1000 associated genes with at least 3 associated genes belonging to gene lists were included in the enrichment analysis. Electronically annotated (IEA) BPs were excluded from the enrichment analysis. *P*-values of enriched pathways and BPs were corrected using the default multiple-hypotheses testing method (g:SCS) of gProfileR; those with an adjusted *P*-value <0.05 were retained.

#### Ploidy

Gene set enrichment analysis was performed using GSEA software^61^. Genes were ranked using the sign of log2(fold change) * -log10(*P*-value) and analyzed using the pre-ranked option. Gene sets from MSigDB, pathways from Reactome, and biological processes from Gene Ontology were included in the analysis. Gene sets larger than 200 were excluded. Significantly enriched pathways were corrected with FDR and only genes with q-value <0.01 were retained.

#### Integrative

Genes were ranked by the number of patients with a mutation, focal copy number events or SV fusion event in a given gene. Pathway analysis was conducted using gProfileR with the following parameters *‘ordered_query* = *TRUE, exclude_iea* = *TRUE, min_set_size* = *5, max_set_size* = *1000, min_isect_size* = *2, max_p_value* = *0.05 and, correction_method* = *“analytical”’*. The GMT file was retrieved from gProfileR on March 12, 2019 and included gene sets from Gene Ontology and Reactome.

### Cytoscape network visualization

#### Pathway enrichment

Visualization of enriched pathways and biological processes (BPs) was generated with the Enrichment Map plugin of Cytoscape^62,63^. Enriched pathways and BPs are organized into a network, in which similar pathways or BPs cluster together. Nodes represent an enriched pathway or BP; node size is proportional to the number of genes associated with the node; and node colors correspond to the Shh-MB subgroup in which they are enriched. Nodes that are connected by an edge have shared genes in common. Edge thickness is proportional to the number of shared genes among the connected nodes and edges having a Jaccard and Overlap coefficient combined greater than 0.66 were shown.

#### Fusion network

A curated list of Tier 1 exon-exon and salvaged SV fusions was input into Cytoscape. This network was further filtered to include fusions hubs with a minimum of 5 events, as well as their first-degree partners. The network was then manually curated to focus on fusions with SV and/or validation support.

### Methylation array arm level copy number analysis

The copy number was inferenced using methylation arrays (Illumina Infinium HumanMethylation450 BeadChips). Copy number segmentation was performed from genome-wide methylation arrays using the conumee package (v0.99.4) in the R statistical environment (v3.2.3)^64,65^. Arm level gains or losses were identified using GISTIC and manually curated by visual inspection of whole-genome profiles.

### Identification of promoter methylation responsive genes

The MethylMix R Bioconductor package^32^ was used to identify potential cancer driver genes affected by hypomethylation or hypermethylation changes (i.e., looking for anti-correlation between methylation level and gene expression levels across samples). Probes were annotated^66^ and filtered to only include regions within 1500 bp of the transcription start site. Promoter probes that correlated were grouped as a probe set, then each promoter probe or probe set was considered per gene. Methylation clusters based on a mixture model were then identified for each probe or probe set. These were further filtered based on the following criteria: (1) remove promoter probe-gene pairs if one of the methylation clusters has less than 5% of the samples and for pairs with two methylation clusters, (2) pairs were filtered out if the difference of the mean methylation value between the 2 groups was <0.25 and (3) if the difference of the mean expression value between the two groups was <0.75. The pairs were further ranked according to a score defined as diff mean * diff exp (difference computed between the 2 extreme clusters). *Z*-score expression values were used to compute the mean expression differences mentioned above.

### Illustrations

Oncoprint landscape figures were generated in R (v3.5.1) using the ComplexHeatmap (v2.0.0) library^67^. Gene mutation, fusion summary lollipop type figures were generated using ProteinPaint^68^. Circos plots were generated in CIRCOS^69^ (v0.69).

### Reporting summary

Further information on research design is available in the Nature Research Reporting Summary linked to this article.

## Supplementary information

Supplementary Information

Description of Additional Supplementary Files

Supplementary Data 1

Supplementary Data 2

Supplementary Data 3

Supplementary Data 4

Supplementary Data 5

Supplementary Data 6

Supplementary Data 7

Supplementary Data 8

Supplementary Data 9

Reporting Summary

## Data Availability

The RNA-seq data generated from this study has been deposited in the European Genome-Phenome Archive (EGA) database under the accession code EGAD00001006305. The published medulloblastoma RNA-seq data referenced in this study is available in the European Genome-Phenome Archive (EGA) database under the accessions EGAD00001004435, EGAD00001001899, and EGAD00001004958. The referenced GTEx normal cerebellum RNAseq controls were acquired from the NCBI public repository phs000424.v6.p1. The Affymetrix SNP 6.0 data referenced during the study are available in the Gene Expression Omnibus (GEO) under the accession GSE37385. The whole-genome sequencing data referenced during the study are available in EGA under the accessions EGAD00001003125 and EGAD00001004347. The Illumina 450k methylation data referenced during the study are available in GEO under the accession GSE85218. The Affymetrix HuGene 1.1 ST data referenced during the study are available in GEO under the accessions GSE85218 and GSE37384. There were multiple databases used for annotation and filtering referenced in this study. These include the Exome Aggregation Consortium [https://gnomad.broadinstitute.org/downloads], the NHLBI-ESP project [https://esp.gs.washington.edu/drupal/], the Kaviar Genomic Variant Database [http://db.systemsbiology.net/kaviar/#:~:text=Kaviar%20Genomic%20Variant%20Database%20%7C%20SNP,and%20frequency%20of%20observed%20variants.], the Haplotype Reference Consortium [http://www.haplotype-reference-consortium.org/], the Greater Middle East Variome [http://igm.ucsd.edu/gme/], the Brazilian Genomic Variants Database [http://abraom.ib.usp.br/], RADAR [http://rnaedit.com/], and GENCODE (v19) [https://www.gencodegenes.org/human/release_19.html]. All the other data supporting the findings of this study are available within the article and its supplementary information files and from the corresponding author upon reasonable request. A reporting summary for this article is available as a Supplementary Information file.
