# Supplementary material for: The transcriptional landscape of Shh medulloblastoma
Source: Nat Commun. 2021 Mar 19;12:1749. doi: 10.1038/s41467-021-21883-0 (PMC7979819; doi:10.1038/s41467-021-21883-0)
Supplement: Supplementary file 1 — Supplementary Information [file 41467_2021_21883_MOESM1_ESM.pdf]

The Transcriptional Landscape of Shh Medulloblastoma

Skowron *et el.*

Supplementary Information

Contents:

Supplementary Figure 1 - Copy number responsive Genes in GISTIC regions.

Supplementary Figure 2 - Transcriptional landscape of aneuploid tumors.

Supplementary Figure 3 - Fusion calling overview.

Supplementary Figure 4 - Fusion landscape.

Supplementary Figure 5 - Copy number alterations in fusion hubs.

Supplementary Figure 6 - Genes with promoter DNA methylation anticorrelated with change in gene expression across Shh-MB.

Supplementary Figure 1

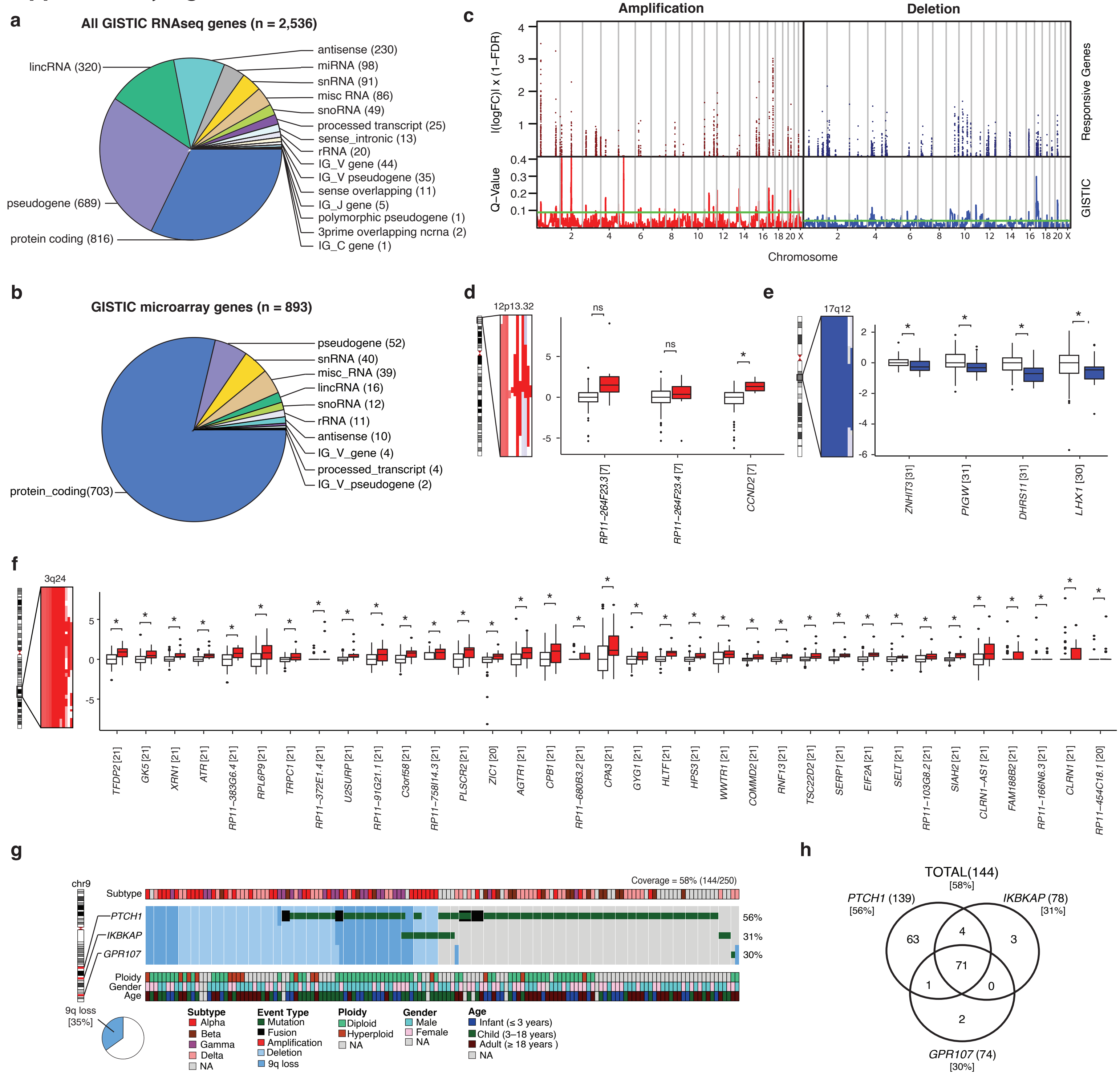

Supplementary Figure 1 - Copy number responsive Genes in GISTIC regions. a–b Gene type distribution of all genes found within GISTIC regions using (**a**) RNA-seq and (**b**) within the subset of genes found only on microarrays. **c** Distribution of significant copy number responsive genes (top) GISTIC peaks (bottom). Scores are calculated based on the absolute value of the fold change in GISTIC region expression and the copy number responsive analysis FDR P-value. The threshold for GISTIC significant peaks is shown as a horizontal line. **d–f** Expression difference between copy number neutral and aberrant states in GISTIC regions. **d** All recurrently amplified genes in 12p13.32. **e** Copy number responsive genes in the 17q12 deletion GISTIC region. **f** Copy number responsive genes in 3q24 recurrently gained GISTIC region. Genes annotated with an asterisk are significantly copy number responsive (Kruskal-Wallis rank sum test FDR adjusted < 0.05). Please refer to Supplementary Data 5 for exact *P*-values. The GISTIC region copy number segments are shown to the left of each graph. Numbers in square brackets denote the number of patients detected with copy number alterations. Expression of each gene was normalized by the expression median of the neutral copy number state. **g–h** Oncoprint summary of (**g**) *PTCH1*, *IKBKAP*, *GPR107* events and (**h**) their overlaps. Lower and upper hinges in the boxplot correspond to the first and third quartiles while the centre line represent the median. The upper and lower whisker extend from the nearest hinge to the smallest/largest value at most 1.5 times the interquartile range. Points outside this range are outliers and are plotted individually.

Supplementary Figure 2

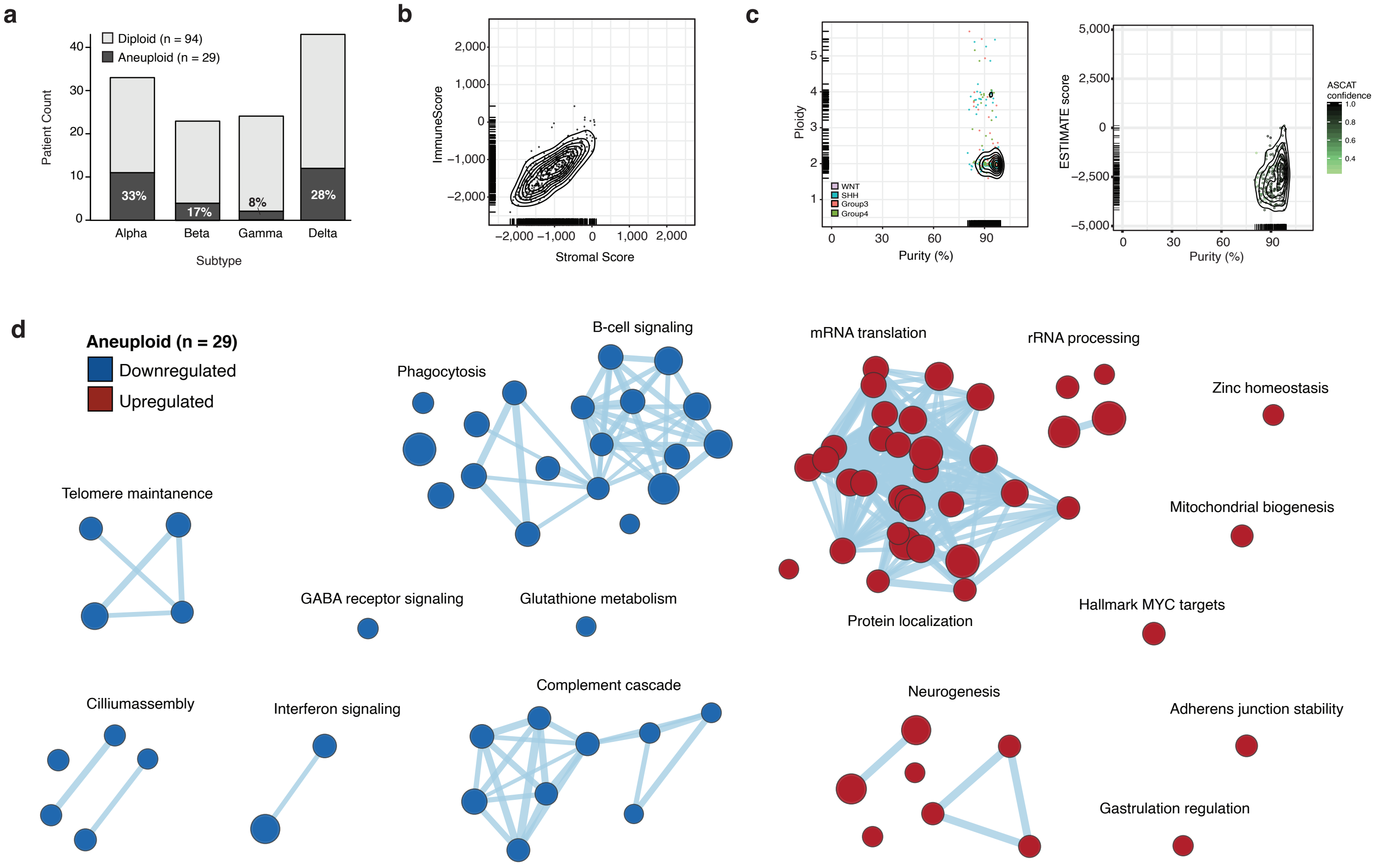

Supplementary Figure 2 - Transcriptional landscape of aneuploid tumors. **a** Number of diploid and aneuploid tumors across Shh-MB subtypes. **b** Tumor Purity assessment using RNA-seq ESTIMATE SNP6 Stromal and Immune scores. Scores below zero denote higher tumor purity. **c** SNP6 calculated purity compared to ploidy (left) and RNA-seq derived ESTIMATE score (right). **d** GSEA enrichment map of differentially expressed genes in aneuploid (n = 29) compared to diploid (n = 94) Shh-MB tumors (FDR < 0.01). Node size is proportional to the number of genes and edge weight represents the number of shared genes between each gene set. The color represents where the pathway was found to be overexpressed; either diploid (blue) or aneuploid tumors (red).

Supplementary Figure 3

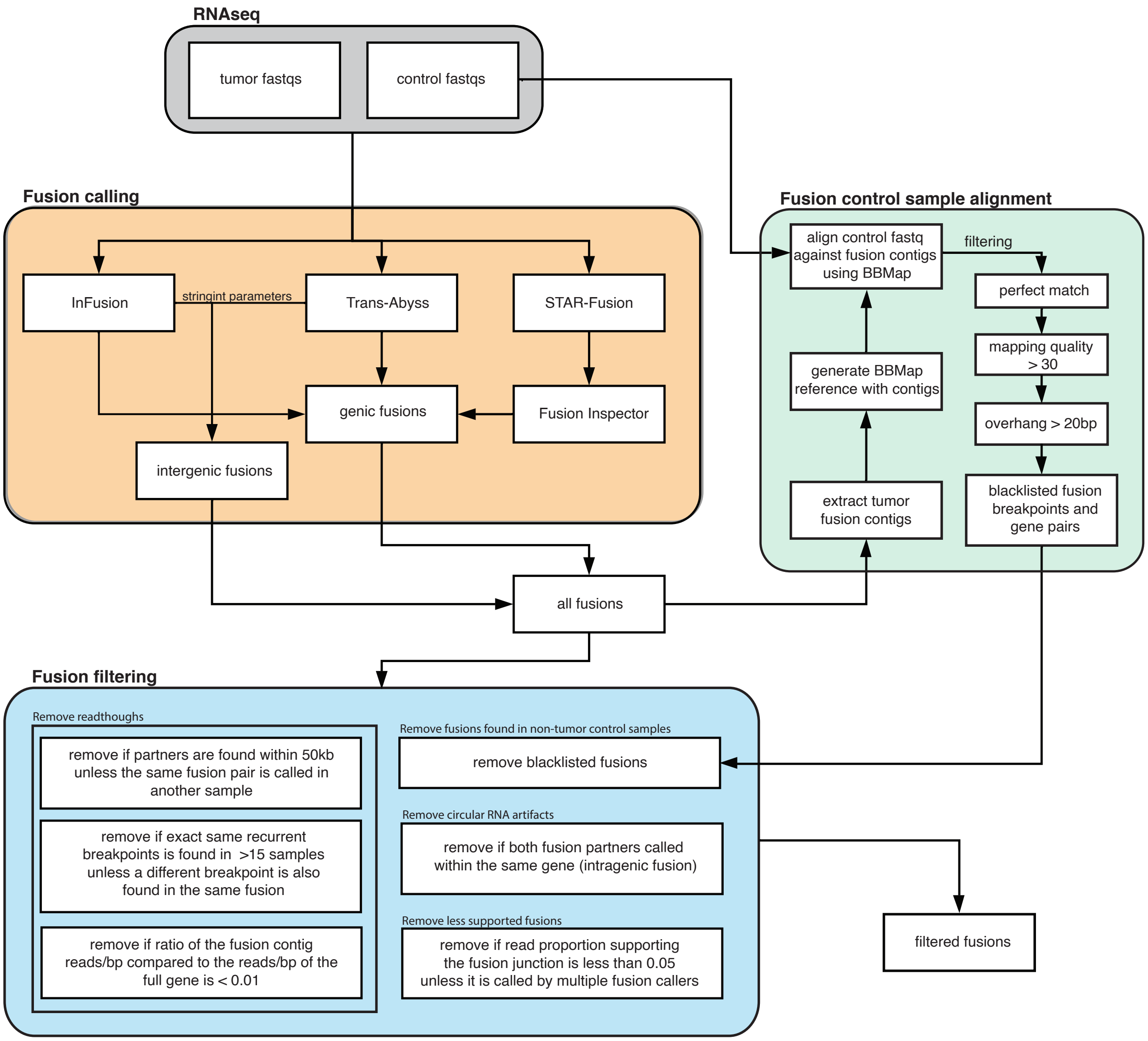

Supplementary Figure 3 - Fusion calling overview. Flowchart of the fusion calling method (n = 250) using InFusion, STAR-Fusion and Trans-ABYSS. Fusion contigs matching reads in the GTEx and Biotech control samples (n = 51) were filtered, as well as any read through, subclonal, and intragenic fusions.

Supplementary Figure 4

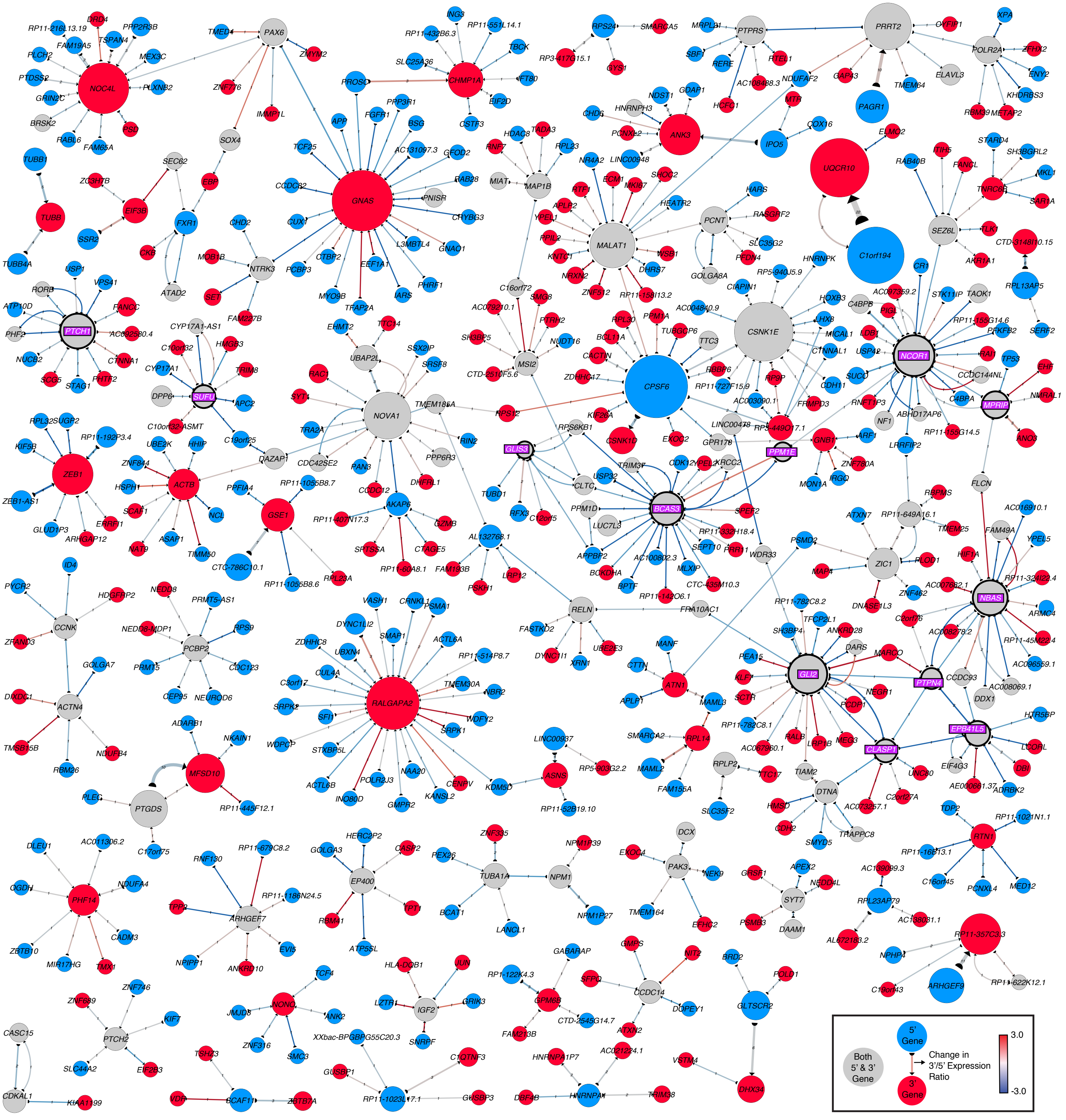

Supplementary Figure 4 - Fusion landscape. Exon-exon fusion network in Shh-MB. The color represents orientation of the gene (5′ is blue , 3′ is red, and both is grey), while the size of the node is proportional to the recurrence of the gene. The color of the line shows fold change difference in gene 3′ or 5′ expression ratio fusion positive compared to negative patients, while line thickness is proportional to the recurrence. Fusion hubs supported by structural variants are encircled with highlighted gene names.

Supplementary Figure 5

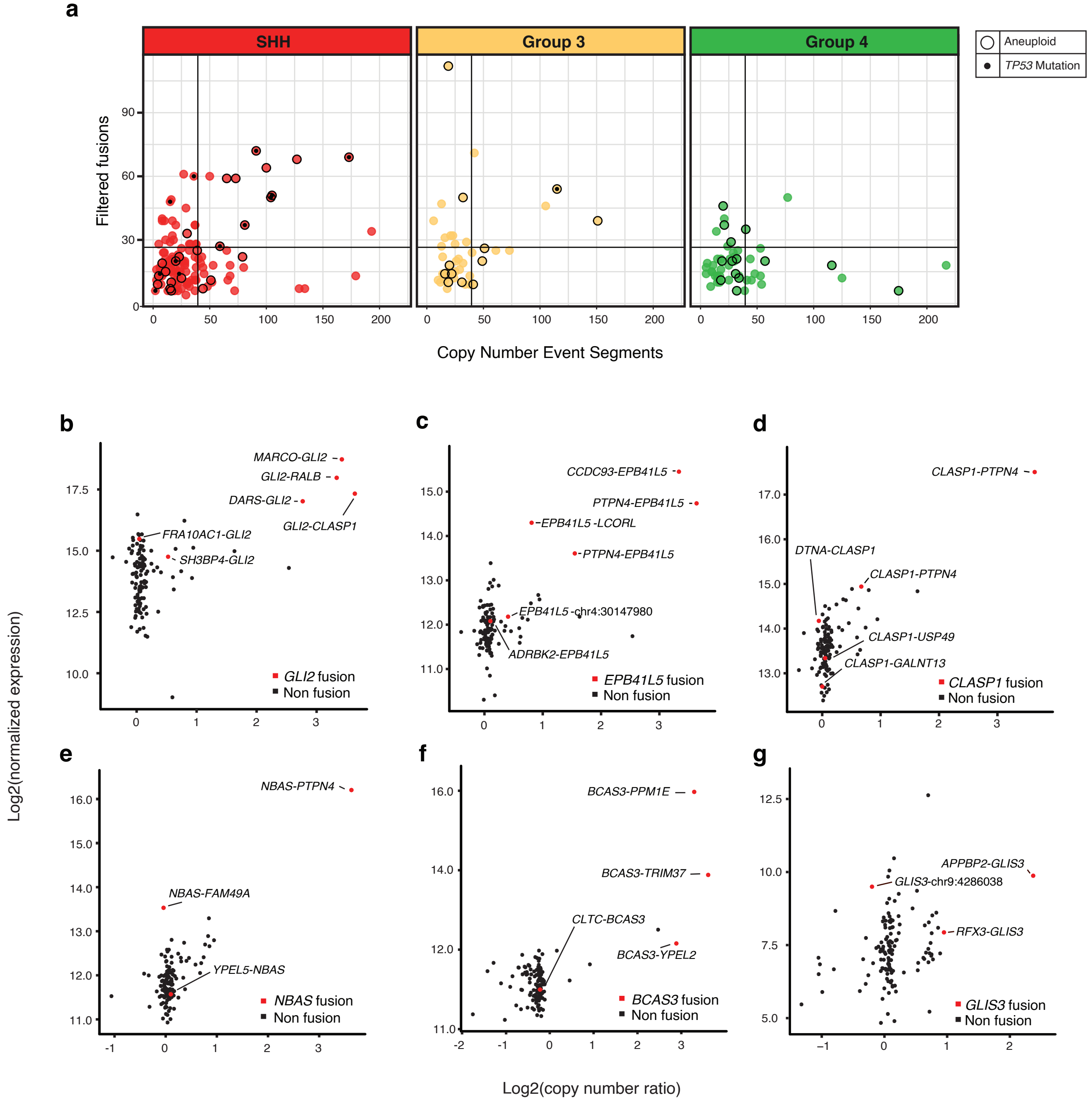

Supplementary Figure 5 - Copy number alterations in fusion hubs. a Correlation between the number of copy number segments and the number of fusions in Shh-MB (n = 250), Group 3-MB (n = 56), and Group 4-MB (n = 61). Aneuploidy status is shown across all subgroups. *TP53* mutation status is only known for Shh-MB patients. **b-g** Correlation between expression and copy number for (**b**) *GLI2*, (**c**) *EPB41L5*, (**d**) *CLASP1*, (**e**) *NBAS*, (**f**) *BCAS3*, and (**g**) *GLIS3*. Fusion tumors are indicated in red. The fusion with the most read support in its respective patient is shown.

Supplementary Figure 6

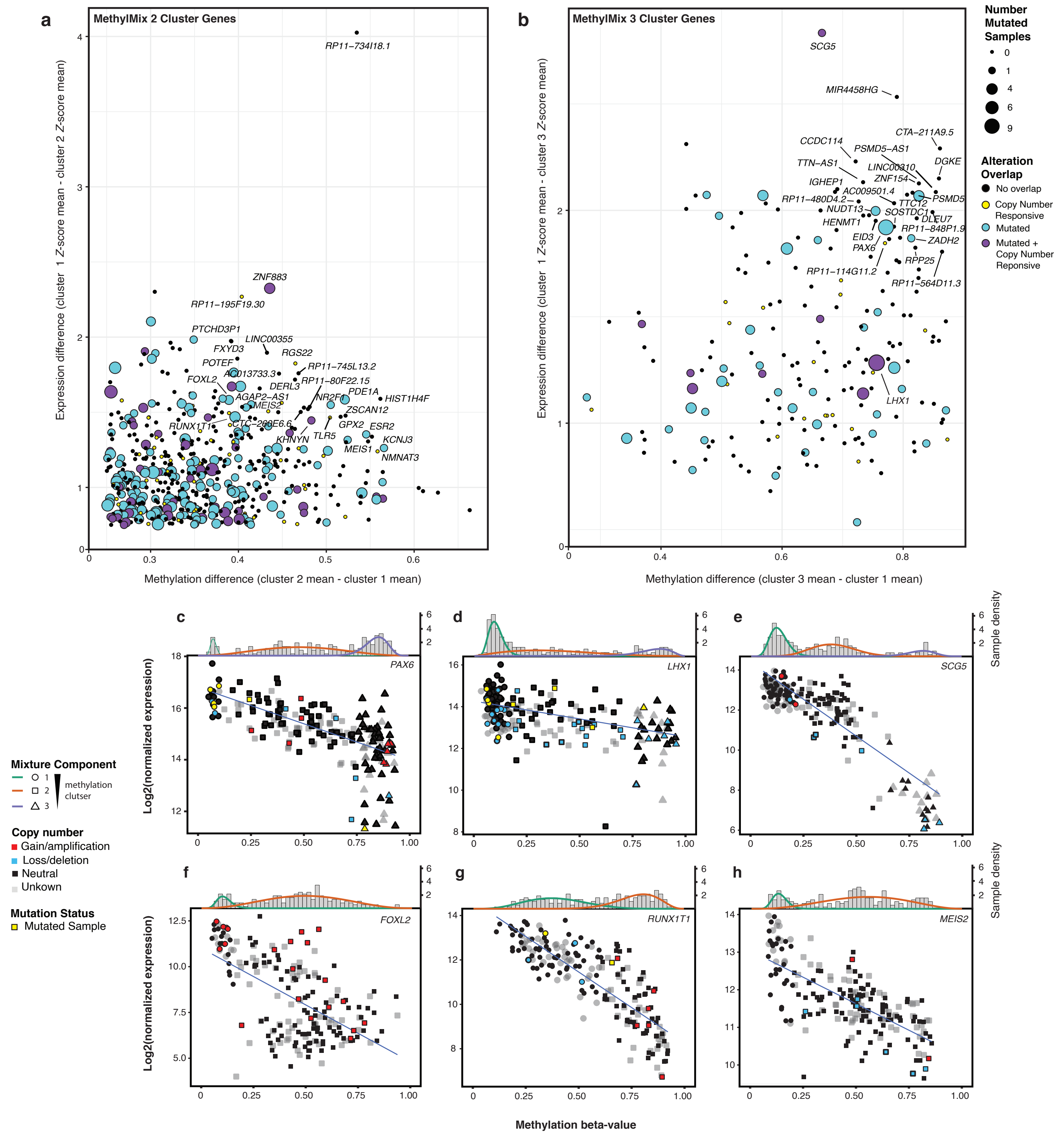

Supplementary Figure 6 - Genes with promoter DNA methylation anticorrelated with change in gene expression across Shh-MB. a–b Scatterplot showing the mean difference in expression and methylation between samples with **a** two methylation clusters discovered by MethylMix, and **b** between samples from three methylation clusters. The size of the points is proportional to the number of patients with mutations found in the corresponding gene. Points are colored by their overlap in mutation events and if they were found to be copy number responsive. **c–h** Correlation of gene expression and DNA methylation in genes identified by MethylMix for (**c**) *PAX6*, (**d**) *LHX1*, (**e**) *SCG5*, (**f**) *FOXL2*, (**g**) *RUNX1T1*, (**h**) *MEIS2*. The methylation clusters are highlighted in a histogram above each scatterplot and are represented by different shapes in the bottom plot. The point border and fill colors correspond to the copy number and mutation state of the given gene, respectively for each Shh-MB sample.
