# Supplementary material for: The transcriptional landscape of Shh medulloblastoma
Source: Nat Commun. 2021 Mar 19;12:1749. doi: 10.1038/s41467-021-21883-0 (PMC7979819; doi:10.1038/s41467-021-21883-0)
Supplement: Supplementary file 2 — Description of Additional Supplementary Files [file 41467_2021_21883_MOESM2_ESM.pdf]

Description of Supplementary Data

Supplementary Data 1 - Sample annotation. Sample clinical annotation and overlap with published Affymetrix SNP 6.0 arrays, 450K methylation arrays, whole genome sequencing (WGS), and Affymetrix HuGene 1.1 expression arrays.

Supplementary Data 2 - Genes driving Shh-MB subtypes. a Top 10% of subtype-relevant genes (NMI). **b–e** Differential gene expression of (**b**) Shh-α versus Shh-β, Shh-γ, Shh-δ. (**c**) Shh-β versus Shh-α, Shh-γ, Shh-δ. (**d**) Shh-γ versus Shh-α ,Shh-β, Shh-δ. (**e**) Shh-δ versus Shh-α, Shh-β, Shh-γ. DESseq2 was used for differential expression, with log2(fold change) ≥ 2 and FDR ≤ 0.05.

Supplementary Data 3 - Mutations. a Recurrence of mutations across the four subtypes. Subgroup enrichment scores in columns B–E range from red (more enrichment) to blue (less enrichment). Exact test was used to measure significance of subtype enrichment. **b** Detailed mutation information for functionally significant genes. MutSigCV was used to call gene mutation significance.

Supplementary Data 4 - Shh-MB GISTIC peaks. Significant GISTIC peak information (adjusted P-value < 0.25). Number of genes found by microarray and RNAseq annotations are indicated for each region in columns H–J. All the detected genes and copy number responsive genes are in columns M, N.

Supplementary Data 5 - Copy number responsive genes. a All significant copy number genes detected in focal (≤ 12Mb) copy number aberrations. **b** All significant copy number genes detected in broad (> 12Mb) copy number aberrations. **c** All significant copy number genes considering both focal and broad copy number aberrations. Kruskal-Wallis rank sum test was performed on each gene to determine if the gene copy number state corresponds with a significant difference in expression (FDR < 0.05).

Supplementary Data 6 - Fusions. a Recurrence of fusions across the four subtypes. Subgroup enrichment scores in columns b-e range from red (more enrichment) to blue (less enrichment). Exact test was used to measure significance of subtype enrichment (column P). Fold change difference in whole gene expression (fused vs. non-fused) is indicated in columns R–U. Difference between 3′ or 5′ gene expression ratios (fused vs. non-fused) is indicated in Z–AC. Significant values are bolded. **b** Filtered fusion events detected by STAR-Fusion, Trans-ABYSS, and INFUSION fusion callers. Events validated by SNP 6.0 or WGS are indicated in columns O and P. **c** Filtered fusion with structural variant support detected by STAR-Fusion, Trans-ABYSS, and INFUSION fusion callers. **d** Fusion flag descriptions. **e** SV fusions amount pairwise comparisons using two-sided Wilcox rank-sum test.

Supplementary Data 7 - Integrative analysis. Integration of methylation responsive, copy number aberrant, copy number responsive, and significantly mutated genes found in Shh-MB.

Supplementary Data 8 - Mutually exclusive genes. a–e Co-occurring and mutually exclusive gene pairs in (**a**) Shh-α, (**b**) Shh-β, (**c**) Shh-γ, (**d**) Shh-δ, and (**e**) across all subtypes. The DISCOVER package and a two-sided Fisher Exact test were used to calculate significance with FDR < 0.01. **f** Summary of significant mutually exclusive genes from (**a–e**). ME - mutual exclusivity, CO - co-occurrence.

Supplementary Data 9 - MethylMix 2 and 3 cluster genes. a Genes containing two methylation clusters significantly correlated with a change in gene expression. **b** Genes containing three methylation clusters significantly correlated with a change in gene expression.
