# Supplementary material for: The transcriptional landscape of Shh medulloblastoma
Source: Nat Commun. 2021 Mar 19;12:1749. doi: 10.1038/s41467-021-21883-0 (PMC7979819; doi:10.1038/s41467-021-21883-0)
Supplement: Supplementary file 12 — Reporting Summary [file 41467_2021_21883_MOESM12_ESM.pdf]

nature research

Corresponding author(s): Michael D. Taylor
Hiromichi Suzuki

Last updated by author(s): Sep 10, 2020

Reporting Summary

Nature Research wishes to improve the reproducibility of the work that we publish. This form provides structure for consistency and transparency in reporting. For further information on Nature Research policies, see our Editorial Policies and the Editorial Policy Checklist.

Statistics

For all statistical analyses, confirm that the following items are present in the figure legend, table legend, main text, or Methods section.

n/a	Confirmed	
[ ]	[x]	The exact sample size (*n*) for each experimental group/condition, given as a discrete number and unit of measurement
[ ]	[x]	A statement on whether measurements were taken from distinct samples or whether the same sample was measured repeatedly
[ ]	[x]	The statistical test(s) used AND whether they are one- or two-sided<br>*Only common tests should be described solely by name; describe more complex techniques in the Methods section.*
[ ]	[x]	A description of all covariates tested
[ ]	[x]	A description of any assumptions or corrections, such as tests of normality and adjustment for multiple comparisons
[ ]	[x]	A full description of the statistical parameters including central tendency (e.g. means) or other basic estimates (e.g. regression coefficient) AND variation (e.g. standard deviation) or associated estimates of uncertainty (e.g. confidence intervals)
[ ]	[x]	For null hypothesis testing, the test statistic (e.g. *F*, *t*, *r*) with confidence intervals, effect sizes, degrees of freedom and *P* value noted<br>*Give P values as exact values whenever suitable.*
[x]	[ ]	For Bayesian analysis, information on the choice of priors and Markov chain Monte Carlo settings
[x]	[ ]	For hierarchical and complex designs, identification of the appropriate level for tests and full reporting of outcomes
[ ]	[x]	Estimates of effect sizes (e.g. Cohen's *d*, Pearson's *r*), indicating how they were calculated

Our web collection on statistics for biologists contains articles on many of the points above.

Software and code

Policy information about availability of computer code

Data collection	No software was used to collect data.
Data analysis	STAR (v2.5.1b),GATK (v3.8.0), EBCall (v0.2.1), MutSigCV (v1.41), Affymetrix Power Tools (v1.18.2), ASCAT (v2.4.3), GISTIC (v2.0), STAR-Fusion (v0.8.0), Infusion (v0.7.3), bbmap (v37.33), bedtools (v2.27.1), HTseq (v0.10.0), Burrows-Wheeler Aligner (v0.7.8), Genomon-SV (v0.4.1), DELLY2 (v0.7.5), biobambam (v0.0.148), PyMOL (v2.3), UCSF Chimera (v1.13.1), GSEA (v4.0.1), Cytoscape (v3.7.2), CIRCOS (v0.69), R packages: SNFtool (v2.2.0), DESeq2 (v1.24.0), Gviz (v1.18.2), ggseqlogo (v0.1), DISCOVER (v1.1.0), gProfileR (v0.6.8), conumee (v0.99.4), MethylMix (v2.14.0), ComplexHeatmap (v2.0.0)

For manuscripts utilizing custom algorithms or software that are central to the research but not yet described in published literature, software must be made available to editors and reviewers. We strongly encourage code deposition in a community repository (e.g. GitHub). See the Nature Research guidelines for submitting code & software for further information.

Data

Policy information about availability of data

All manuscripts must include a data availability statement. This statement should provide the following information, where applicable:

- Accession codes, unique identifiers, or web links for publicly available datasets
- A list of figures that have associated raw data
- A description of any restrictions on data availability

Data used by this study is available in the European Genome-Phenome Archive (EGA) and the Gene Expression Omnibus (GEO): RNA-seq (EGAD00001004435 [https://ega-archive.org/datasets/EGAD00001004435], EGAD00001001899 [https://ega-archive.org/datasets/EGAD00001001899], EGAD00001004958 [https://ega-archive.org/datasets/EGAD00001004958], EGAD00001006305 [https://ega-archive.org/datasets/EGAD00001006305]), whole genome sequence (EGAD00001003125 [https://ega-archive.org/datasets/EGAD00001003125], EGAD00001004347 [https://ega-archive.org/datasets/EGAD00001004347]), Affymetrix

SNP 6.0 (GSE37385 [https://www.ncbi.nlm.nih.gov/geo/query/acc.cgi?acc=GSE37385]), Illumina 450k methylation (GSE85218 [https://www.ncbi.nlm.nih.gov/geo/query/acc.cgi?acc=GSE85218]), Affymetrix HuGene 1.1 ST (GSE85218 [https://www.ncbi.nlm.nih.gov/geo/query/acc.cgi?acc=GSE85218], GSE37384 [https://www.ncbi.nlm.nih.gov/geo/query/acc.cgi?acc=GSE37384]). GTEX normal cerebellum controls were acquired from (phs000424.v6.p1) [https://www.ncbi.nlm.nih.gov/projects/gap/cgi-bin/study.cgi?study_id=phs000424.v6.p1]. Databases used for annotation and filtering include: Exome Aggregation Consortium [https://gnomad.broadinstitute.org/downloads], NHLBI-ESP project [https://esp.gs.washington.edu/drupal/], Kaviar Genomic Variant [http://db.systemsbiology.net/kaviar/#:~:text=Kaviar%20Genomic%20Variant%20Database%20%7C%20SNP,and%20frequency%20of%20observed%20variants.], Haplotype Reference Consortium [http://www.haplotype-reference-consortium.org/], Greater Middle East Variome [http://igm.ucsd.edu/gme/], Brazilian Genomic Variants [http://abraom.ib.usp.br/], RADAR [http://rnaedit.com/], and GENCODE (v19) [https://www.gencodegenes.org/human/release_19.html].

Field-specific reporting

Please select the one below that is the best fit for your research. If you are not sure, read the appropriate sections before making your selection.

- [x] Life sciences
- [ ] Behavioural & social sciences
- [ ] Ecological, evolutionary & environmental sciences

For a reference copy of the document with all sections, see nature.com/documents/nr-reporting-summary-flat.pdf

Life sciences study design

All studies must disclose on these points even when the disclosure is negative.

Sample size	Sample size was determined by the availability of the human samples. We used all sequencing data we have.
Data exclusions	No data was excluded from this analysis
Replication	No experiments were performed in this exploratory study.
Randomization	All available Sonic Hedgehog Medulloblastoma patients, as part of the Medulloblastoma Advanced Genomics International Consortium, were selected for this genomic study and sequenced using RNAseq. Where applicable, only the Sonic Hedgehog subtypes (Shh-α, Shh-β, Shh-γ, Shh-δ) were used as covariates for analysis.
Blinding	This is an exploratory study. The same analysis scripts were used for all the tumor samples. Investigators were not blinded to the cancer type and/or subtypes during the interpretation of the results. All available Sonic Hedgehog medulloblastomas from the Medulloblastoma Advanced Genomics International Consortium were used in this study.

Reporting for specific materials, systems and methods

We require information from authors about some types of materials, experimental systems and methods used in many studies. Here, indicate whether each material, system or method listed is relevant to your study. If you are not sure if a list item applies to your research, read the appropriate section before selecting a response.

Materials & experimental systems

n/a	Involved in the study	
x		Antibodies
x		Eukaryotic cell lines
x		Palaeontology and archaeology
x		Animals and other organisms
	x	Human research participants
x		Clinical data
x		Dual use research of concern

Methods

n/a	Involved in the study	
x		ChIP-seq
x		Flow cytometry
x		MRI-based neuroimaging

Human research participants

Policy information about studies involving human research participants

Population characteristics	Samples were collected at diagnosis after informed consent was obtained from subjects as part of the Medulloblastoma Advanced Genomics International Consortium. Research participants were patients with confirmed diagnosis of medulloblastoma at local centers. All cases used in this study were primary treatment-naive Sonic Hedgehog medulloblastomas. This cancer comprises four molecular subtypes, 26% Shh-α (adolescents), 21% Shh-β (babies with a poor prognosis), 16% Shh-γ (babies with a good prognosis), and 37% Shh-δ (adults). The male-to-female ratio was approximately 1:1.6.
Recruitment	Patients were recruited by investigators from each local center. There is no bias of recruitment because patiens are not prescreened. Potential self-selection bias or other biases were not identified.
Ethics oversight	The samples were collected under a SickKids Research Ethics Board (REB) approved protocol REB: 0020020238 "Formation of Nervous System Tumor Tissue Bank at the Hospital for Sick Children".

Note that full information on the approval of the study protocol must also be provided in the manuscript.
